# AI spring and its regulation discourse: a bibliometric study of trends in literature

**DOI:** 10.3389/frai.2026.1762748

**Published:** 2026-05-07

**Authors:** Katja Debelak, Primož Pevcin, Rok Hržica

**Affiliations:** Faculty of Public Administration, University of Ljubljana, Ljubljana, Slovenia

**Keywords:** accountability, AI spring, artificial intelligence, bibliometric analysis, governance, regulation

## Abstract

**Introduction:**

This study was prompted by the rapid acceleration of AI capabilities in the trans former era since 2018 and the concurrent regulatory shift that elevated legal accountability and public governance to central policy and research priorities. It contributes by treating 2018–2025 as a distinct governance regime in which transformer-enabled capability scaling and foundation models shifted AI gov ernance debates toward enforceable accountability architectures.

**Methods:**

The study maps the regulation–accountability–public governance nexus as an operational problem: which accountability forums dominate, which regulatory instruments anchor in the field, and which public-administration mechanisms remain underdeveloped. Using multiple queries in the Web of Science Core Collection, validated with Scopus, and analyzed with Bibliometrix and complementary science-mapping techniques, the study examines publication trends, influen tial contributors and outlets, collaboration networks, and citation and thematic structures.

**Results:**

Publication output increases sharply after 2022, aligning with major regulatory milestones such as the EU AI Act. Results show a strong European concentration, with European actors serving as central hubs in collaboration networks and indicate that 2018–2021 publications form a foundational intel lectual core. The field is anchored in legally oriented concepts (law, transparency, governance, accountability, data protection), while themes such as legitimacy, institutional logics, and rights operationalization remain underdeveloped.

**Discussion:**

Despite growing interdisciplinarity, thematic fragmentation persists, highlighting the need for stronger integration across legal scholarship, public administration, and tech nical AI research, and providing a focused basis for future research and policy agendas.

## 1 Introduction

Artificial Intelligence (AI) has experienced several ups and downs since its emergence in the 1950s (Nilsson, 2009). These fluctuations have generated several waves of AI; some authors refer to these as seasons (Haenlein and Kaplan, 2019). Currently, we are experiencing a new boom, often labeled the AI Boom or AI Spring (Cruz and Treisman, 2018), initiated by the development of the transformer architecture. The pioneering work introducing this deep learning architecture, known as the transformer, was published in December 2017 by a Google-sponsored group of authors (Vaswani et al., 2017).

The advantage of transformers is that they do not have recurrent units, which reduces training time (Hochreiter and Schmidhuber, 1997). Transformers have improved previous machine translation methods (Zhao et al., 2023) and have since been applied in many areas, including natural language processing, reinforcement learning, and audio processing (Chen et al., 2021; Monastirsky et al., 2023).

As noted, the introduction of the transformer model initiated the current wave of AI applications. This supports using 2018 as the starting year for new discussions and research related to AI (Singh and Mahmood, 2021; Yadav, 2024). This is further emphasized by the increasing number of results in the Web of Science database, where publications related to AI and transformers have risen steadily since 2018, especially after 2020 (Web of Science, 2025).

The AI boom gained international prominence after 2020, corresponding to the age of integration and ethics, when AI’s integration into business, public services, and everyday technology became more profound (Kashefi et al., 2024; Microsoft, 2026). This has led to growing concerns about bias, privacy, and the societal impacts of AI, moving beyond the technological aspects of AI utilization, and resulting in the current hype surrounding AI (Mora-López et al., 2025). Moreover, selecting 2018 as the starting point is important for the uniqueness and value added by the research, as it marks the transition from experimental to widespread deployment of AI, when organizations began integrating AI into core processes and global attention to ethical, regulatory, and societal implications intensified following developments such as GDPR and growing concerns about algorithmic bias. This year also represents the inflection point preceding the post-2020 AI boom, allowing the review to capture both the foundations and acceleration of AI’s modern integration and societal impact. By grounding the timeframe in this socio-technological shift, the study offers a unique perspective that goes beyond technical evolution and situates AI development within the broader context of ethics, governance, and real-world adoption.

AI systems increasingly influence public sector decision-making, administrative processes, and regulatory environments. Scholars and policymakers have begun to examine how automated decision systems interact with legal principles, public accountability, and governance mechanisms. This has expanded research across law, public administration, political science, computer science, and ethics, creating a rapidly growing but fragmented body of literature. Issues such as algorithmic accountability, regulatory compliance, liability, transparency obligations, and risk governance have become central to discussions of AI in democratic societies, particularly within the European context, where regulatory initiatives such as the EU AI Act and OECD guidelines are shaping international debates on responsible AI. Despite this growth, the field lacks an evidence-based, reproducible map of who drives the discourse, where it is published, how its intellectual foundations are structured, and which themes are consolidating versus underdeveloped, limiting cumulative theory building and actionable guidance for public governance.

Given the rapid growth and interdisciplinary nature of this field, a systematic mapping of the scientific landscape is necessary to understand its evolution, structure, and research gaps. Bibliometric analysis offers a quantitative and reproducible method for examining publication trends, identifying influential authors and institutions, analyzing collaboration networks, and revealing the conceptual and intellectual structures of a research domain (Cobo et al., 2011; Zupic and Čater, 2015; Aria and Cuccurullo, 2017; Donthu et al., 2021). This mapping is particularly timely because the post-2018 transformer era and the regulatory turn have reshaped both the volume and content of scholarship, yet the resulting knowledge structure has not been systematically consolidated. Applying this approach to literature at the intersection of AI, regulation, legal accountability, and public governance enables identification of how scholarly attention has shifted alongside technological and regulatory developments.

The objective of this study is to provide a reproducible bibliometric mapping of research on artificial intelligence regulation, legal accountability, and public governance from 2018 to 2025. Specifically, the study (i) quantifies publication growth, (ii) identifies influential authors, institutions, countries, and outlets, (iii) maps collaboration and citation structures, and (iv) diagnoses thematic clusters and gaps. The core contribution is an evidence-based account of how the field is structured in the transformer era, enabling clearer integration across legal scholarship, public administration, and technical AI research, and providing a focused basis for future research and policy agendas.

The research questions follow a standard bibliometric approach, moving from mapping the size and dynamics of the field to explaining its structure and implications. RQ1 establishes the growth trajectory of scientific production in the 2018–2025 transformer-era window. RQ2 and RQ3 identify where the discourse is produced and curated by locating influential contributors and primary publication outlets. RQ4 reveals the field’s intellectual foundations and their evolution through citation and co-citation structures. RQ5 assesses diffusion and cross-jurisdictional knowledge accumulation by mapping collaboration patterns. RQ6 identifies thematic consolidation around accountability, transparency, ethical principles, and regulatory frameworks. RQ7 synthesizes these empirical mappings to specify persistent research gaps and translate them into priorities for future scholarship and policy design.

Accordingly, this study is guided by the following research questions:

RQ1: What are the global trends in scientific production on artificial intelligence regulation and public governance between 2018 and 2025 (van Raan, 2014; Zupic and Čater, 2015; Donthu et al., 2021; Passas, 2024)? This establishes the field’s production trajectory in the transformer era.

RQ2: Who are the most influential authors, institutions, and countries contributing to the literature on AI regulation, legal accountability, and governance (Van Eck and Waltman, 2010; Cobo et al., 2011; Aria and Cuccurullo, 2017)? This identifies where influence and capacity are concentrated.

RQ3: Which journals and publication outlets disseminate scholarship on AI governance, administrative law, and regulatory oversight (Moher et al., 2009; Zupic and Čater, 2015; Chen, 2017; Donthu et al., 2021)? This locates the main venues that curate and legitimize scholarship.

RQ4: What are the dynamics of citations, including the most cited documents, co-citation structures, and temporal citation patterns (Van Eck and Waltman, 2010; Cobo et al., 2011; Donthu et al., 2021; Passas, 2024)? This maps the intellectual base and its temporal evolution.

RQ5: How are scientific collaborations structured among authors, institutions, and countries in this field (Aria and Cuccurullo, 2017; Deng et al., 2021)? This captures diffusion mechanisms through collaboration structures.

RQ6: What emerging themes can be identified in the literature, particularly regarding accountability, transparency, ethical principles, and regulatory frameworks (Cobo et al., 2011; Zupic and Čater, 2015; Donthu et al., 2021; Passas, 2024)? This diagnoses thematic consolidation and emerging governance priorities.

RQ7: What research gaps persist within the study of AI regulation and public governance, and how can these gaps inform future scholarly and policy agendas (van Raan, 2014; Chen, 2017; Donthu et al., 2021)? This translates mapping results into a future research and policy agenda.

This paper’s novelty is not a general mapping of AI governance or AI ethics. It is a theory-relevant delimitation to the transformer era governance problem, defined by a capability shift (foundation-model scale and generalization) (Vaswani et al., 2017; Bommasani et al., 2021), rapid diffusion into high-stakes administrative decision processes (Oswald, 2018; Kuziemski and Misuraca, 2020; Alon-Barkat and Busuioc, 2023), and a regulatory turn toward risk-based, lifecycle obligations and enforceable compliance architectures. Existing bibliometric studies typically aggregate multiple governance waves, which risks conflating pre-transformer ethical debates with the contemporary regulation-accountability problem (Gao et al., 2024; Yuan et al., 2024). Therefore, we isolate transformer era scholarship (2018–2025) at the intersection of AI regulation, legal accountability, and public governance, rather than surveying AI governance broadly. This design allows us to map how accountability is being operationalized through regulation, including the concepts and venues that dominate, the collaboration hubs that shape agenda-setting, and the thematic fragmentation that limits cumulative theory building.

## 2 Materials and methods

### 2.1 Literature search, data collection and preprocessing

A bibliometric review was selected for this study because it is well suited to broad research domains and large datasets. This approach enables the analysis of publication trends, intellectual structures, and collaboration networks—insights that cannot be obtained through meta-analyses or traditional systematic reviews, which are more appropriate for narrow topics with smaller, homogeneous evidence bases (Cobo et al., 2011; Zupic and Čater, 2015; Aria and Cuccurullo, 2017; Donthu et al., 2021).

The bibliometric analysis was conducted using R version 4.5.1 (Ihaka, 2022; The R Foundation, 2025) and the Bibliometrix package developed by Aria and Cuccurullo (2017). Bibliometrix provides a comprehensive framework for quantitative science mapping and performance analysis, enabling extraction, preprocessing, and visualization of bibliographic data within a single reproducible workflow. The methodological workflow consisted of five main steps:

(1) data collection,
(2) data cleaning and preprocessing,
(3) descriptive performance analysis,
(4) science mapping, and
(5) cross-validation using an independent bibliographic database (Scopus).

Bibliographic records were retrieved from the Web of Science (WoS) due to its reliability for citation-based analyses and broad coverage of peer-reviewed literature. As discussed by Hržica et al. (2025), WoS remains one of the most authoritative and widely used databases for assessing the scientific landscape, due to its structured metadata, historical depth, and robust citation-tracking functionalities. Its comprehensive indexing facilitates longitudinal analyses while maintaining high standards of quality control (Li et al., 2018). Furthermore, WoS supports accurate retrieval and analysis of Open Access publications at the article level (Pranckutė, 2021). In addition, WoS provides a curated and consistently indexed corpus with standardized metadata and export formats, supporting reproducible bibliometric workflows and improving comparability across records. This is especially important for science mapping and text-based analyses that rely on consistent abstracts and keyword fields (Zupic and Čater, 2015; Mongeon and Paul-Hus, 2016).

The search strategy targeted literature at the intersection of artificial intelligence, regulation, legal accountability, and public governance. It relied on Boolean keyword matching in WoS, with conceptual coverage ensured by explicitly including synonymous and closely related terms within each thematic group (Bramer et al., 2018). The query also incorporated policy- and institution-related terms (European Union, EU AI Act, OECD, Council of Europe, international law) to ensure that the retrieved literature explicitly engages with regulatory frameworks and formal governance structures, rather than focusing solely on technical or abstract discussions of AI. This design choice intentionally centers the corpus on governance-oriented research anchored in an identifiable institutional context.

However, including EU- and OECD-related terms may bias retrieval toward European and Western policy environments and may underrepresent governance debates from other regions. This potential limitation is inherent in the study’s conceptual focus on AI regulation and accountability frameworks that have been most prominently institutionalized through the EU AI Act process and OECD/Council of Europe governance initiatives. Therefore, the results should be interpreted as representing the structured and formalized strand of AI governance literature, rather than the full global body of work on AI in the public sector.

No temporal restriction was applied in the database search. However, the retrieved corpus naturally covered the period 2018–2025, as no relevant papers were indexed before 2018. This period corresponds to the emergence of transformer-based AI architecture at the end of 2017, marking a new technological and conceptual phase in AI development. Research on AI governance, legality, and public sector impact expanded rapidly only after this shift, while earlier literature reflects prior technical paradigms that would dilute the analytical focus. Therefore, focusing on the 2018–2025 period provides a coherent and contemporary evidence base aligned with ongoing regulatory debates.

The final query executed in WoS was: [(“artificial intelligence” OR “AI regulation” OR “automated decision-making”) AND (“legal uncertainty” OR “regulatory gap” OR “accountability” OR “compliance” OR “liability” OR “transparency” OR “rule of law” OR “subjectivity”) AND (“public sector” OR “public administration” OR “digital government” OR “public law” OR “administrative law” OR “governance” OR “public policy”) AND (“European Union” OR “EU AI Act” OR “OECD” OR “Council of Europe” OR “international law”)]. This query structure includes four conceptual blocks—AI, regulatory and legal accountability concepts, public governance context, and institutional or policy framework—to ensure that the retrieved records explicitly address AI governance and accountability in the public sector context.

This query retrieved 209 articles covering the period from 2018 to 2025.

The records were exported from Web of Science in plain text format and imported into R using the Bibliometrix package. Data cleaning and preprocessing included:

(1) removal of duplicates,
(2) harmonization of author names and affiliations,
(3) standardization of country names and management of affiliations per record,
(4) consolidation and standardization of keywords across the dataset.

To ensure transparency and reproducibility (PRISMA-inspired), the key details are reported as follows:

- Database: Web of Science
- Query string: [(“artificial intelligence” OR “AI regulation” OR “automated decision-making”) AND (“legal uncertainty” OR “regulatory gap” OR “accountability” OR “compliance” OR “liability” OR “transparency” OR “rule of law” OR “subjectivity”) AND (“public sector” OR “public administration” OR “digital government” OR “public law” OR “administrative law” OR “governance” OR “public policy”) AND (“European Union” OR “EU AI Act” OR “OECD” OR “Council of Europe” OR “international law”)]
- Time span: no temporal restrictions
- Number of retrieved and analyzed papers: 209

### 2.2 Bibliometric analysis and science mapping

Descriptive bibliometric analyses were conducted to examine publication output, citation performance, and the geographic distribution of research, including the most productive countries and institutions. This corresponds to the performance analysis stage of bibliometric studies, which assesses research productivity and impact using publication and citation indicators (van Raan, 2014; Donthu et al., 2021). Visualizations were generated using Bibliometrix (Aria and Cuccurullo, 2017) and ggplot2.

Science mapping techniques were applied to explore the intellectual and conceptual structure of the field. Science mapping provides a systematic approach to visualizing relationships, dynamics, and thematic evolution within a research domain (Cobo et al., 2011; Zupic and Čater, 2015; Passas, 2024). The analysis included:

- citation analysis,
- co-citation analysis,
- bibliographic coupling,
- co-word analysis, and
- co-authorship analysis.

Each method captured different dimensions of the intellectual and social structure in science (van Raan, 2014; Deng et al., 2021; Pessin et al., 2022).

Keyword co-occurrence networks and thematic maps were created to identify clusters of related research topics and their interconnections. Network visualizations and summary indicators were used to assess the structural organization and connectivity of the research field, revealing the main thematic clusters and conceptual linkages among them.

All analyses and visualizations were produced in RStudio (Posit, 2025), ensuring reproducibility and transparency of the workflow.

To assess the robustness of the Web of Science (WoS)-based analysis, an independent validation was conducted using the Scopus database. Scopus was selected for validation because, along with WoS, it is one of the two leading, competing citation databases (Zhu and Liu, 2020) and is among the most widely used bibliographic databases, offering broad coverage of peer-reviewed literature across disciplines. Using Scopus for cross-validation ensures that the observed patterns in the WoS dataset are consistent and not artifacts of a single database, thereby enhancing the reliability and generalizability of the bibliometric findings.

The same query was replicated in Scopus using equivalent syntax and the same keyword structure. The Scopus dataset was used exclusively for validation and was not merged with the primary WoS corpus. This approach was chosen for methodological consistency, as merging WoS and Scopus datasets introduces significant duplication and harmonization challenges, including differences in citation indexing, journal coverage, author identifiers, affiliation formatting, keyword normalization, and metadata completeness (Pranckutė, 2021). These differences can distort citation-based indicators and network structures, especially in co-citation and bibliographic coupling analyses, where small inconsistencies in reference indexing can lead to artificial fragmentation of networks.

Accordingly, WoS was retained as the primary analytical dataset because of its structured citation metadata and established suitability for citation-based science mapping, while Scopus was used as an independent benchmark to test whether the dominant patterns observed in WoS are reproducible across a competing indexing system. The design choice prioritizes internal consistency and robustness of bibliometric structures over maximum coverage.

The validation procedure involved comparing the WoS and Scopus datasets along five dimensions:

(1) overall publication volume and annual growth,
(2) the most productive countries,
(3) the most productive and influential authors,
(4) the leading journals in the field, and.
(5) the most frequent keywords and thematic descriptors.

Because WoS and Scopus are the two leading and complementary databases, demonstrating that key bibliometric patterns appear consistently in both sources provides strong evidence that the findings are not database-specific artifacts. These five dimensions cover the most sensitive aspects of database variability, making the cross-database comparison an adequate and widely accepted robustness check in bibliometric research (Archambault et al., 2009).

## 3 Results

This section presents the results of three complementary analyses, each supported by visualizations. First, descriptive performance analyses summarize publication productivity, citation impact, and the geographic distribution of research. Second, science mapping visualizations reveal the intellectual and conceptual structure of the field, highlighting thematic clusters and relationships among research topics. Third, trend analyses illustrate the temporal evolution of key themes and keywords. Together, these analyses provide a comprehensive overview of the development, structure, and evolution of research on artificial intelligence regulation and public governance.

### 3.1 Descriptive performance analysis plots—metrics of productivity, impact, and basic distribution

The descriptive performance analysis provides an overview of research productivity, impact, and distribution across countries, years, and authors. This analysis identifies the main contributors to the field and highlights general publication and citation trends that reflect the field’s development and scholarly attention over time.

Regarding the geographic distribution of publications on artificial intelligence regulation and public governance, the analysis shows a clear concentration of research output in high-income, policy-active regions, particularly the United States (49 articles), United Kingdom (37), Italy (36), and Spain (33), followed by Germany (29) and the Netherlands (21). This pattern reflects strong institutional engagement in AI-related governance issues within the European Union and Anglo-American research ecosystems, where debates on regulation, accountability, and ethics have been most prominent (Batool et al., 2025; Bolgouras et al., 2025).

A second tier of contributions includes Portugal (19), Australia (14), Sweden (13), and France (12), indicating broader international interest. Emerging contributions from China (10), Ukraine (9), India (7), and Romania (7) highlight growing participation from outside Western Europe and North America. Overall, the distribution reveals a geographically uneven research landscape, dominated by European and English-speaking countries but gradually diversifying.

The annual volume of publications on artificial intelligence regulation and public governance shows a clear upward trend over the analyzed period. From just 3 publications in 2018 and 2019, output steadily increased to 9 in 2020, 18 in 2021, 19 in 2022, and 26 in 2023, followed by a sharp rise to 45 publications in 2024 and 86 in 2025.

This upward trajectory aligns with the technological transition triggered by transformer models, which led to a marked increase in research addressing regulatory, legal, and governance dimensions of AI. The selected timeframe captures the onset and expansion of the current research cycle, distinct from earlier, pre-transformer periods.

The lower number of publications in 2025 reflects the deliberately narrow scope of the search strategy, which combines AI, legal accountability, public-sector governance, and supranational regulatory frameworks. Consequently, this analysis is intended to characterize structural patterns within a focused policy-relevant domain, rather than to provide statistically generalizable trends across the broader AI literature. Nevertheless, the trend indicates rapidly growing scholarly interest in governance, legal, and accountability aspects of AI, particularly following the release of major regulatory frameworks such as the EU Artificial Intelligence Act and related OECD policy initiatives. The sharp increase after 2022 likely reflects both the maturation of interdisciplinary research on AI regulation and the growing policy relevance of governance frameworks for artificial intelligence.

Average citations per article peak for earlier publication years and decline for more recent years, reflecting the typical citation lag observed in bibliometric studies: older articles have had more time to accumulate citations, while newer publications are still gaining visibility in the literature. Additionally, the rapid growth of the field increases the denominator in the calculation of average citations, contributing to lower values for recent years. These trends are typical in fast-developing research areas such as artificial intelligence regulation and public governance.

Total citations per year peak around 2020–2022, reflecting both the growing volume of publications and the accumulation of citations over time. Earlier years, such as 2018 and 2019, have fewer total citations due to a smaller number of publications, while the slight decline for 2023 to 2025 is likely a result of citation lag, as newer articles have not yet had enough time to accumulate citations. This pattern is consistent with rapidly evolving fields, where both publication output and citation accumulation increase quickly, but citations naturally take time to accumulate.

**Figure 1:**
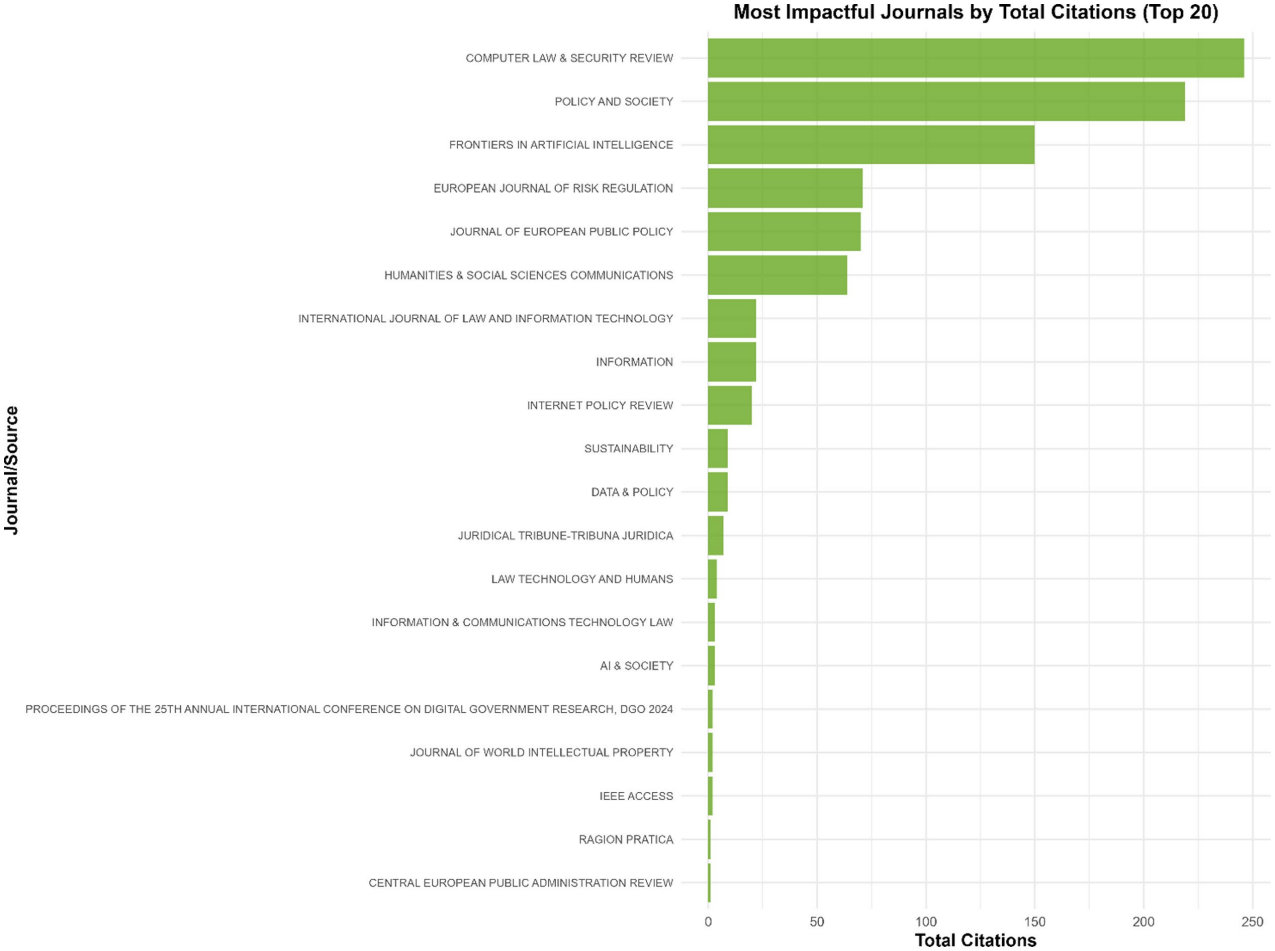
Most impactful journals by total citations.

The analysis of journal impact, measured by total citations (Figure 1), shows that a small group of journals account for a disproportionate share of scholarly influence in the field. Computer Law & Security Review is the most influential, with 246 citations across ten articles. Policy and Society, despite publishing only two articles, has 219 citations and the highest average citation rate. Other journals with notable impact include Frontiers in Artificial Intelligence, European Journal of Risk Regulation, and Journal of European Public Policy, each contributing highly cited work that shapes ongoing debates on AI governance and regulatory frameworks. In contrast, many journals have modest or minimal citation counts, reflecting either recent entry into the field or a narrower topical scope. Overall, the citation distribution highlights how publication influence is concentrated in a limited number of specialized and policy-oriented journals, indicating clear intellectual anchors within the emerging research landscape.

**Figure 2:**
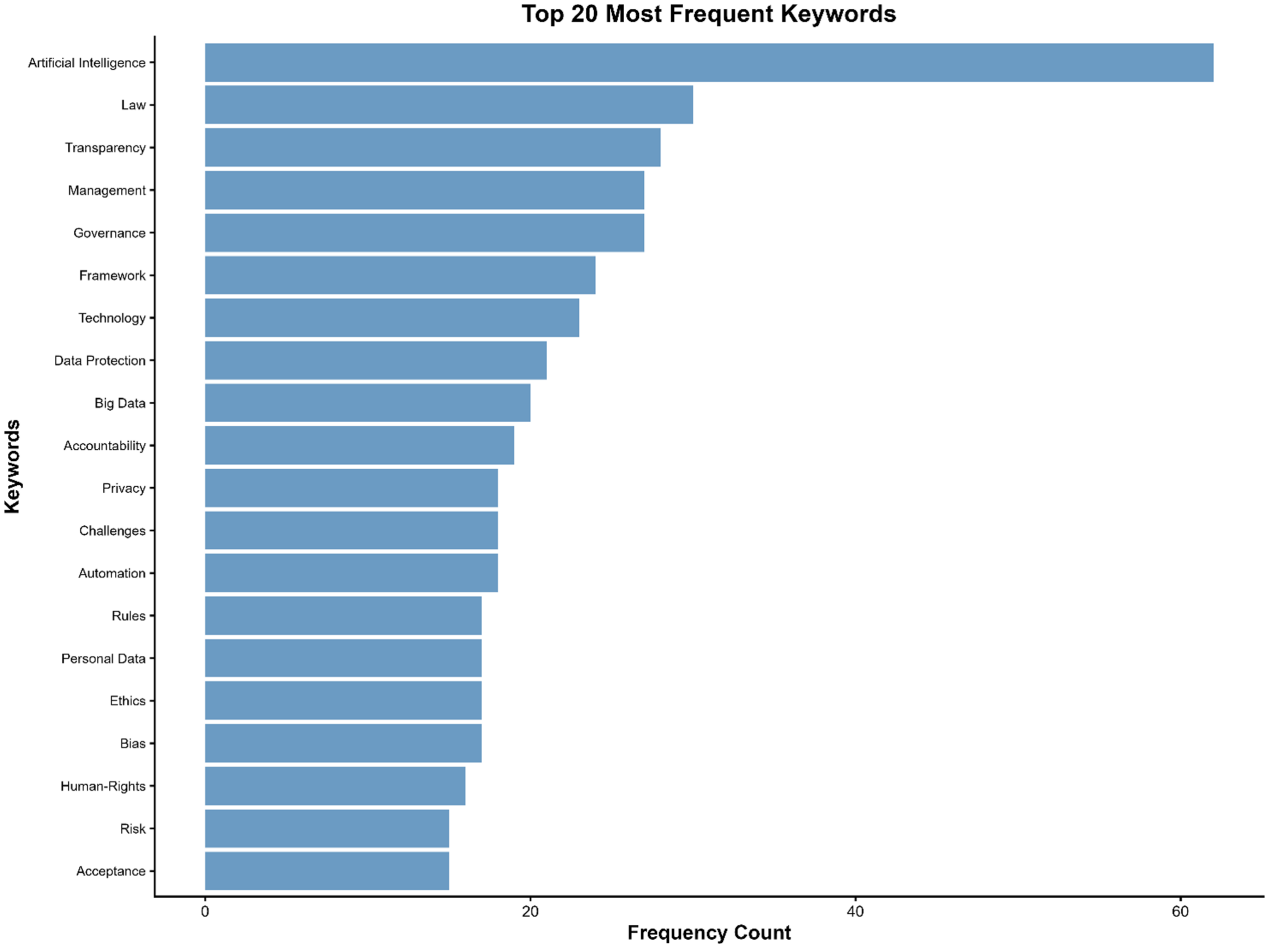
Top 20 words in abstract.

The top 20 most frequent words extracted from the abstract (Figure 2) highlight the dominant topics and themes in the research field. Terms such as AI, legal, data, governance, and intelligence appear most frequently, indicating a strong focus on artificial intelligence and its legal and regulatory implications. Other recurring words, including European, EU, regulation, systems, and transparency, suggest particular attention to European regulatory frameworks and governance structures. This word frequency analysis provides a clear overview of the core concepts and thematic priorities in the literature.

While descriptive performance analyses quantify the number of publications, citation impact, and geographic distribution, they do not capture the conceptual or intellectual relationships among research topics. To address this, the following section uses science mapping techniques to visualize the field’s structure, thematic clusters, and interconnections.

### 3.2 Science mapping/intellectual structure plots—intellectual and conceptual structure, thematic clusters, relationships

Science mapping visualizations offer a detailed view of the conceptual and intellectual structure of a research field. They reveal clusters of related topics, connections among key concepts, and the thematic organization of the literature. These analyses help identify main research themes, explore how topics are interrelated, and uncover the underlying structure of studies on artificial intelligence regulation and public governance. By examining keyword co-occurrence, thematic maps, and collaboration networks, the analysis highlights both prominent topics and their relationships within the field.

Some studies involving AI as a topic have noted the need to explore the dynamics within themes of AI research evolution, specifically by examining its conceptual and intellectual structure (Obreja et al., 2024). Evidence shows significant dynamics in the evolving landscape of generative AI research, where emerging themes increasingly cover a diverse range of themes, from user control and multimodal capabilities to ethical considerations, among others. These research efforts, in addition to shaping the future of generative AI, make the field more versatile, responsible, and socio-economically effective in addressing contemporary challenges (Balasubramaniam et al., 2024). Furthermore, evidence indicates that research output growth is higher in allied and peripheral groups compared to the topical core group of papers, suggesting that AI research now spans a wide range of subject areas (Gupta et al., 2024).

The density-centrality plot visualizes the relevance and development of key research themes. Centrality (X-axis) indicates the importance of a theme within the overall research network, while density (Y-axis) measures its internal development and cohesion. Themes are grouped into four quadrants:

- Motor themes (top-right): high centrality and high density, representing well-developed topics that are central to the field. In this dataset, clear examples of motor themes include Accountability, Human rights, and Impact (centrality > approximately 7 and density > approximately 0.47), which combine strong internal cohesion with interconnections to other topics.
- Basic themes (bottom-right): high centrality but lower density, representing important foundational topics that support multiple research streams but are less internally cohesive. Artificial Intelligence, Governance, Law, Data Protection, and Technology fall in this quadrant, indicating they are widely connected across the field but not are yet tightly developed as coherent subfields.
- Niche themes (top-left): high density but lower centrality, representing specialized and well-developed topics that are more peripheral. Examples include Algorithm and Decision-making, which show strong internal cohesion but fewer links to the broader network.
- Emerging or declining themes (bottom-left): low centrality and low density, representing nascent or less integrated topics. Terms such as Autonomous vehicles, Adoptions, and Rights appear in this area, suggesting limited integration into the main research stream.

**Figure 3:**
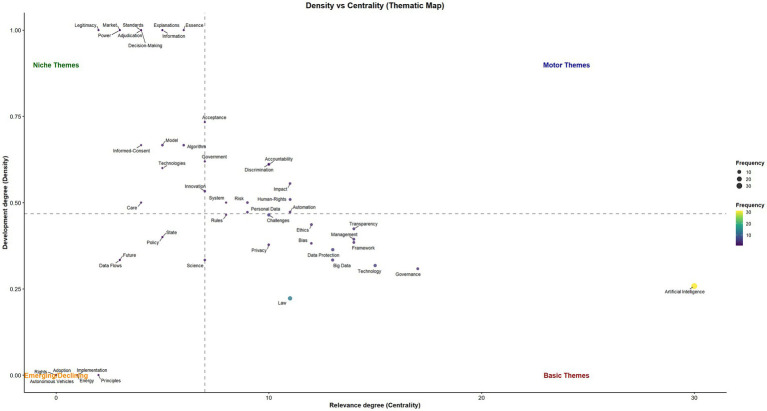
Density vs. centrality (thematic map).

With a median threshold around centrality ≈7 and density ≈0.47, the thematic map shows that the conceptual backbone of the field is driven by broadly relevant, yet variably developed topics positioned as basic themes. Although Artificial Intelligence, Governance, and Law were central search terms, their placement within this quadrant reflects their role as foundational concepts that provide the structural core linking diverse research directions rather than forming tightly cohesive clusters. In contrast, motor themes such as Accountability, Human rights, and Impact represent more focused and well-developed areas of research activity, indicating the emergence of distinct, specialized lines of inquiry built upon these foundational concepts (Figure 3).

The keyword frequency analysis identifies the most recurrent terms in the dataset, providing a quantitative overview of dominant research themes. The twenty most frequent keywords reflect the central conceptual focus of the literature on artificial intelligence regulation and public governance. Artificial intelligence is the most prevalent term (62 occurrences), underscoring its role as the field’s core conceptual anchor. Law (30), Transparency (28), Governance (27), and Management (27) follow closely, highlighting the legal, procedural, and organizational dimensions through which AI-related governance issues are studied.

Complementary terms such as Framework, Technology, Data Protection, Big Data, and Accountability indicate a strong emphasis on regulatory design, technical implications, and ethical oversight. Recurring keywords like Privacy, Automation, Bias, and Ethics point to ongoing debates about fairness, algorithmic integrity, and the protection of individual rights. The appearance of Human Rights (16), Risk (15), and Acceptance (15) further suggests the field’s sensitivity to normative and societal concerns, while Challenges and Impact capture its evaluative and problem-oriented nature.

Overall, the distribution of frequent keywords shows that scholarship in this domain centers on the intersection of AI technologies, legal frameworks, and governance mechanisms, reflecting an interdisciplinary focus that combines regulatory, ethical, and managerial perspectives.

**Figure 4:**
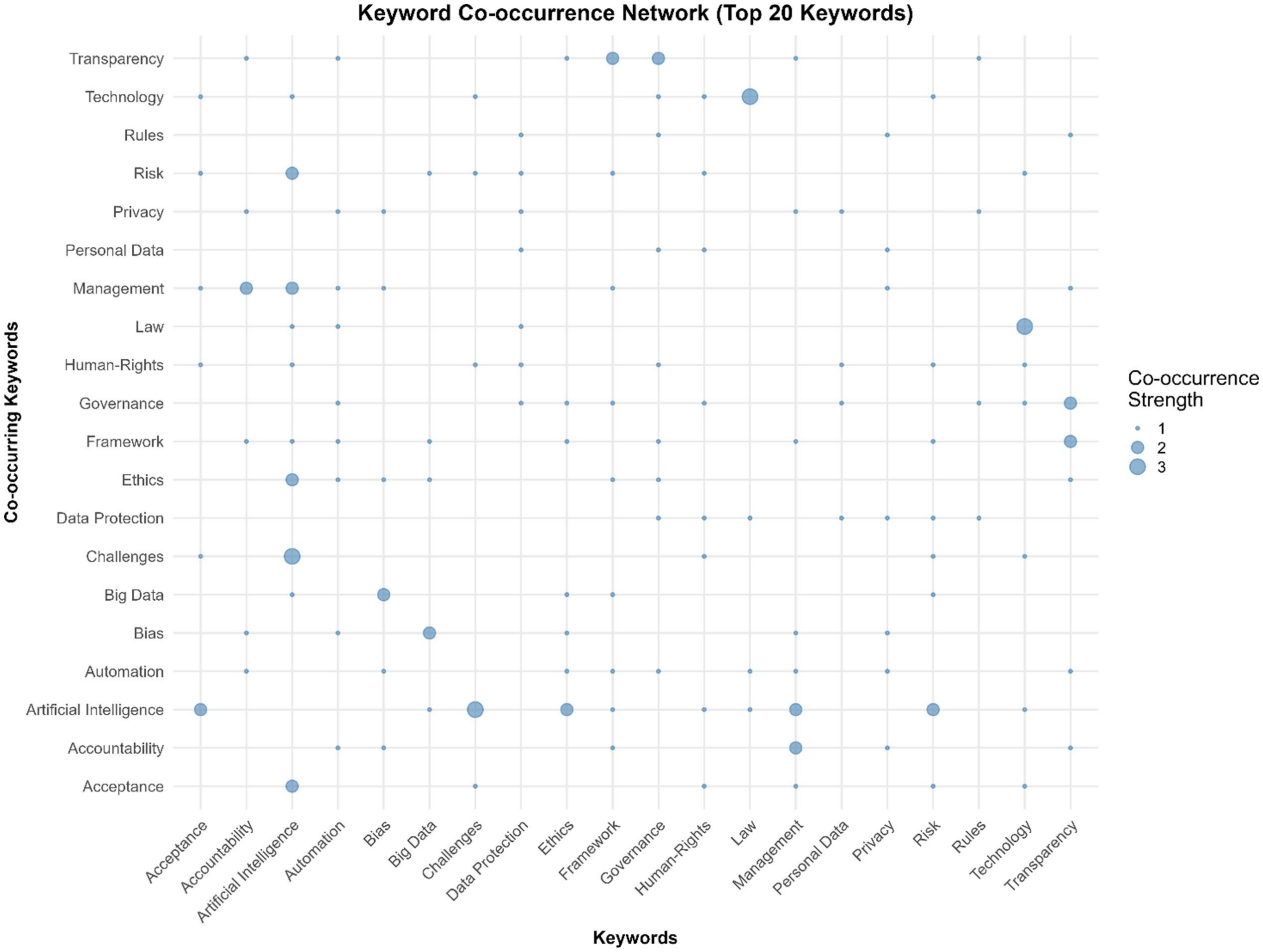
Keyword co-occurrence network (top 20 keywords).

The keyword co-occurrence network (Figure 4) illustrates how key research concepts are interconnected within the literature on artificial intelligence regulation and public governance. Connections between terms indicate their joint appearance in documents, reflecting conceptual proximity and thematic integration. The strongest co-occurrence links are between Artificial Intelligence and Challenges, Law and Technology, and Governance and Transparency, suggesting that much of the discourse centers on practical implementation, regulatory adaptation, and institutional management of AI systems.

The network structure highlights Artificial Intelligence as the dominant hub, connected to clusters of legal (Law, Data Protection), ethical (Ethics, Accountability, Human Rights), and organizational (Governance, Management, Framework) terms. This pattern shows that research in the field is not only technologically focused but also deeply engaged with questions of legitimacy, oversight, and administrative responsibility. Secondary connections among Transparency, Framework, and Accountability further emphasize the growing interest in governance mechanisms that ensure responsible AI use in the public sector (Ghosh et al., 2025).

Overall, the co-occurrence map underscores the interdisciplinary nature of the field, where technical, legal, and ethical dimensions are closely interwoven. This interconnectedness reflects the ongoing effort to bridge conceptual gaps between AI innovation, regulatory frameworks, and governance practices, forming the intellectual foundation of current scholarly debate (Weuts et al., 2025).

**Figure 5:**
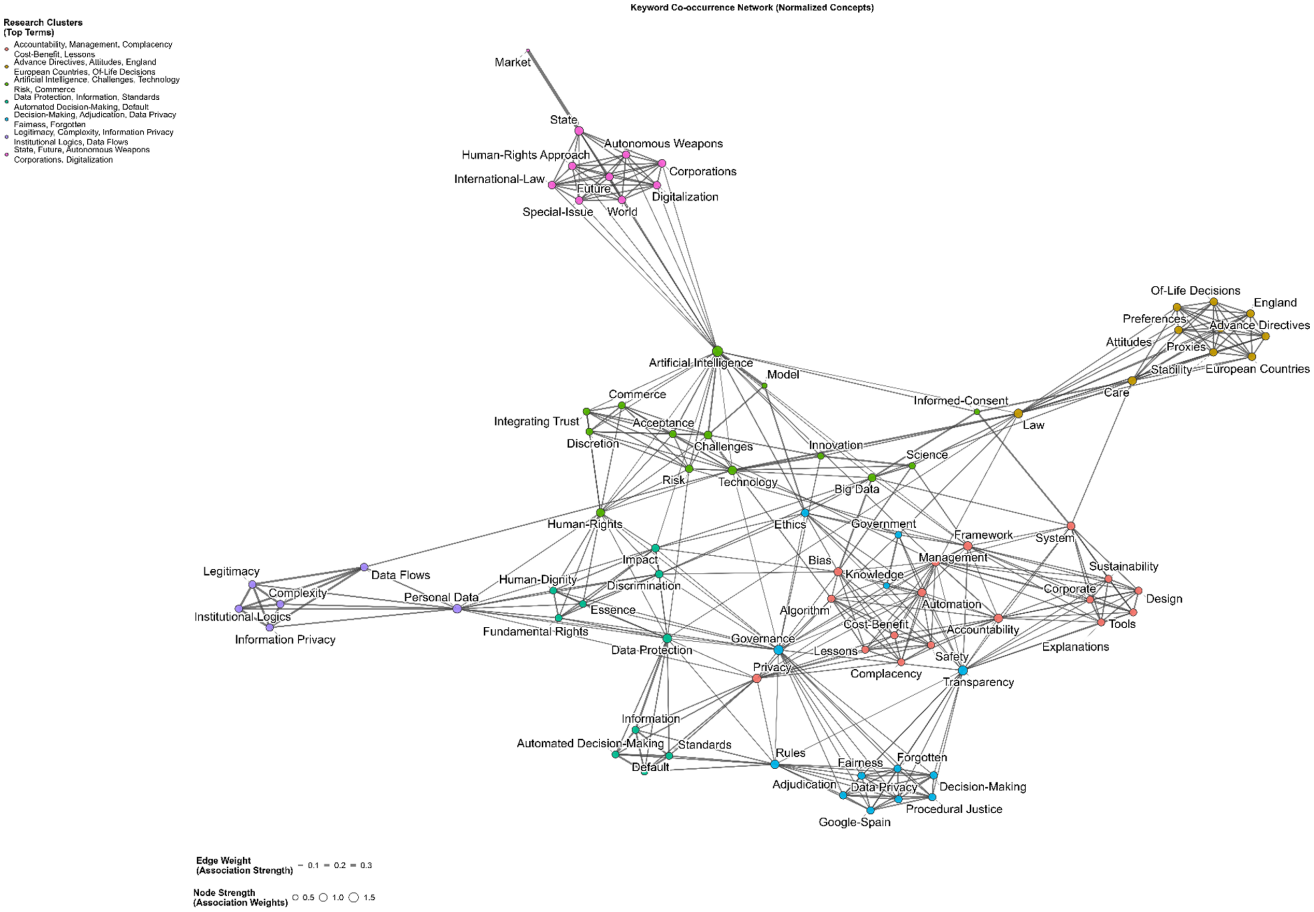
Keyword network normalized.

The normalized keyword network (Figure 5) refines the previous analysis by controlling frequency effects, highlighting the relative strength of conceptual associations among terms. The results show that Law (2.11), Artificial Intelligence (1.80), Governance (1.71), and Data Protection (1.61) form the structural core of the field. These high-strength nodes represent concepts that frequently connect discussions across multiple thematic areas, linking legal, technological, and ethical dimensions of AI governance.

Edges with the highest association weights, such as Law-Technology, Governance-Transparency, Ethics-Automation, and Data Protection-Privacy, indicate a research focus on the regulatory and institutional integration of technical systems. These associations underscore how legal and governance frameworks are increasingly used to implement normative principles such as transparency, fairness, and accountability in the design and oversight of automated decision-making.

Overall, this configuration suggests that scholarship on AI governance in the public sector is converging toward a legally anchored framework, where regulatory and ethical concerns shape how technological systems are designed, implemented, and supervised.

**Figure 6:**
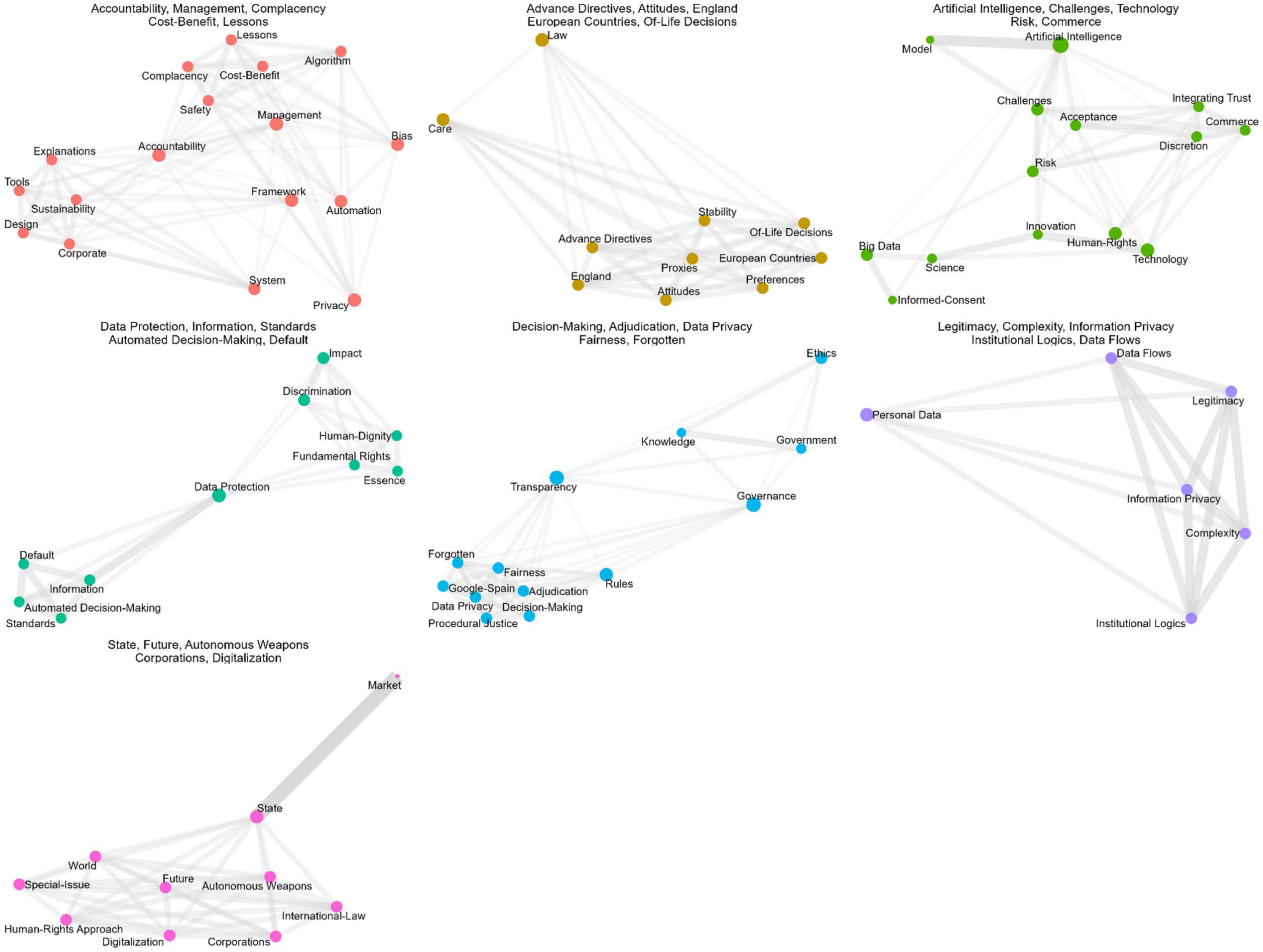
Keyword network faceted.

The faceted keyword network (Figure 6) reveals seven internally cohesive but largely disconnected clusters, indicating that while key concepts such as Law, Governance, and Data Protection form strong local associations, research on AI governance remains thematically fragmented across domain-specific areas. Each cluster represents a distinct strand of inquiry:

- Cluster 1 (“Accountability, Management, Complacency, Cost-Benefit, Lessons”) addresses organizational and managerial aspects of AI governance, focusing on oversight dynamics, cost–benefit analysis, and institutional learning.
- Cluster 2 (“Advance Directives, Attitudes, England, European Countries, Of-life decisions”) covers themes of autonomy, attitudes, and legal practices in specific jurisdictions, suggesting a normative and comparative approach.
- Cluster 3 (“Artificial Intelligence, Challenges, Technology, Risk, Commerce”) centers on the technical and systemic challenges of AI deployment, including risk considerations and commercial dimensions.
- Cluster 4 (“Data Protection, Information, Standards, Automated Decision-Making, Default”) focuses on regulatory standards, data protection mechanisms, and default settings in algorithmic decision systems.
- Cluster 5 (“Decision-Making, Adjudication, Data Privacy, Fairness, Forgotten”) examines procedural, legal, and ethical dimensions of decision systems, emphasizing fairness and privacy in governance contexts.
- Cluster 6 (“Legitimacy, Complexity, Information Privacy, Institutional Logics, Data Flow”) addresses institutional and conceptual issues related to legitimacy, complex systems, and data governance infrastructure.
- Cluster 7 (“State, Future, Autonomous Weapons, Corporations, Digitalization”) explores the geopolitical and strategic implications of AI, intersecting with state power, militarization, and digital transformation.

The faceted keyword network reveals a fragmented thematic structure, characterized by internally cohesive but externally disconnected clusters. Instead of forming a unified research front, the subfields develop in parallel, each advancing distinct conceptual and methodological perspectives. This pattern is consistent with the normalized keyword network, where Law, Artificial Intelligence, Governance, and Data Protection serve as overarching conceptual anchors but do not generate strong cross-cluster linkages. The resulting configuration suggests a plural and loosely integrated research landscape, in which shared regulatory and ethical concerns provide a common vocabulary without yet being consolidated into a fully connected field.

While the faceted keyword network illustrates a fragmented thematic structure, with clusters centered on distinct legal, ethical, and technological domains, the word cloud offers a complementary, integrative perspective. It highlights the most recurrent research themes across the corpus, revealing the conceptual anchors that sustain and connect otherwise dispersed clusters.

The word cloud provides a frequency-based overview of the dominant research themes in the analyzed corpus. The most frequent terms—Artificial Intelligence (62), Law (30), Transparency (28), Governance (27), and Management (27)—indicate that scholarly attention focuses on the regulatory, managerial, and ethical dimensions of AI deployment in public contexts (Aarab et al., 2025). These recurring terms also confirm the prominence of concepts identified in the normalized keyword network, reinforcing the centrality of legally and institutionally grounded discussions.

Mid-frequency terms such as Framework, Technology, Data Protection, Big Data, Accountability, and Automation further highlight a strong alignment between technical innovation and normative governance frameworks, suggesting that research in this domain often bridges computational and juridical discourses.

Lower-frequency but conceptually significant terms, including Human Rights, Fairness, Bias, Privacy, and Risk, reflect ethical and human-centered concerns that complement the legal regulatory core. In contrast, specialized terms such as Autonomous Weapons, Advance Directives, and Algorithmic Decision-Making indicate niche or emerging subtopics at the periphery of the research landscape, consistent with the fragmented structure observed in the faceted keyword network.

Overall, the word cloud corroborates the thematic centrality of AI governance as a legally anchored but still diverse field, where normative principles such as transparency, accountability, and fairness structure the ongoing dialogue between technology and regulation.

**Figure 7:**
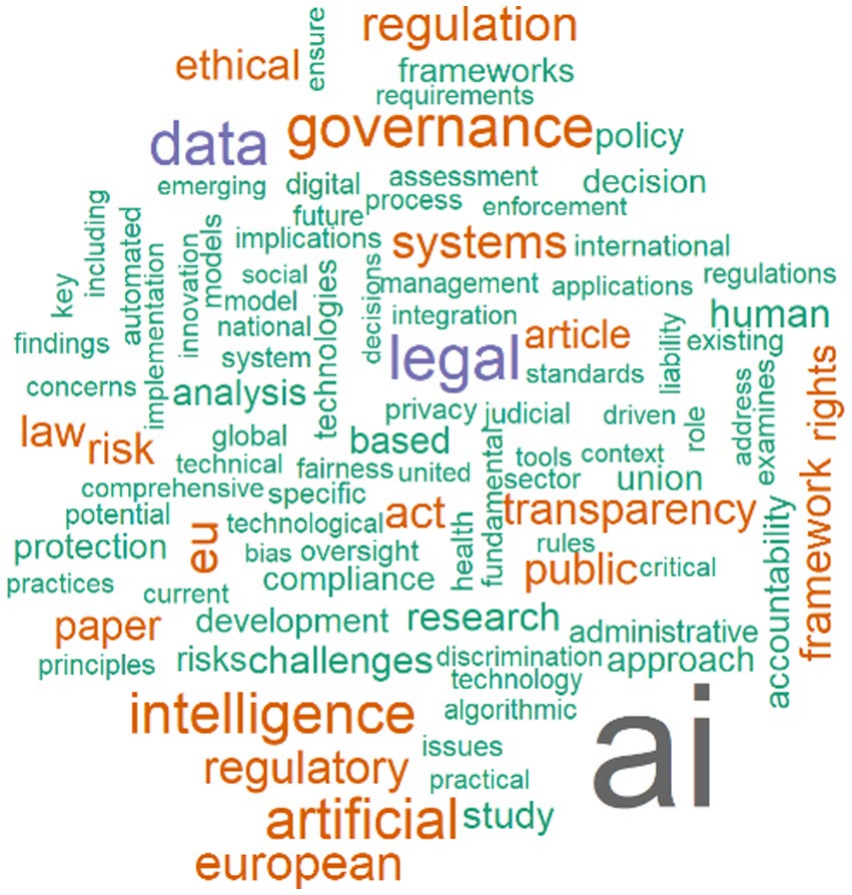
Word cloud abstract.

To complement the keyword-based analysis, the word cloud generated from article abstracts (Figure 7) provides a more nuanced view of how the field articulates its central concerns in textual context. While keywords reveal the formal indexing of concepts, abstract terms reflect the discursive framing and conceptual vocabulary through which scholars discuss AI governance and regulation.

The most frequent terms—AI, legal, data, governance, intelligence, artificial, regulation, and law—underscore the field’s strong juridical-technological orientation (Batool et al., 2025; Hulok, 2025), where the development and oversight of AI systems are framed primarily through regulatory, ethical, and compliance perspectives. The prominence of the EU, European, regulation, and act highlights the European Union’s legislative and policy leadership, particularly regarding the AI Act and data protection frameworks such as the GDPR.

Secondary clusters of high-frequency terms (e.g., transparency, accountability, fairness, ethics, risk, privacy, human rights, discrimination) suggest a strong normative dimension that aligns with principles of responsible and trustworthy AI. Methodological and analytical terms (framework, approach, analysis, assessment, implementation, evaluation) indicate that the literature is not only normative but also increasingly empirical and policy-oriented, focusing on practical mechanisms of governance and oversight.

Additionally, the recurrence of sectoral references such as public, health, healthcare, and administration indicates the importance of AI regulation within the public sector, especially in contexts involving high-stakes decision-making and service delivery. Emerging technical terms like algorithmic, automated, generative, machine learning, and technological innovation reveal the ongoing interplay between technological evolution and regulatory adaptation.

Taken together, the word cloud derived from abstracts shows that scholarly discourse on AI governance is characterized by a legal-regulatory core, an ethical-normative frame, and a growing emphasis on practical governance applications across sectors, with the European regulatory model serving as a key reference point.

**Figure 8:**
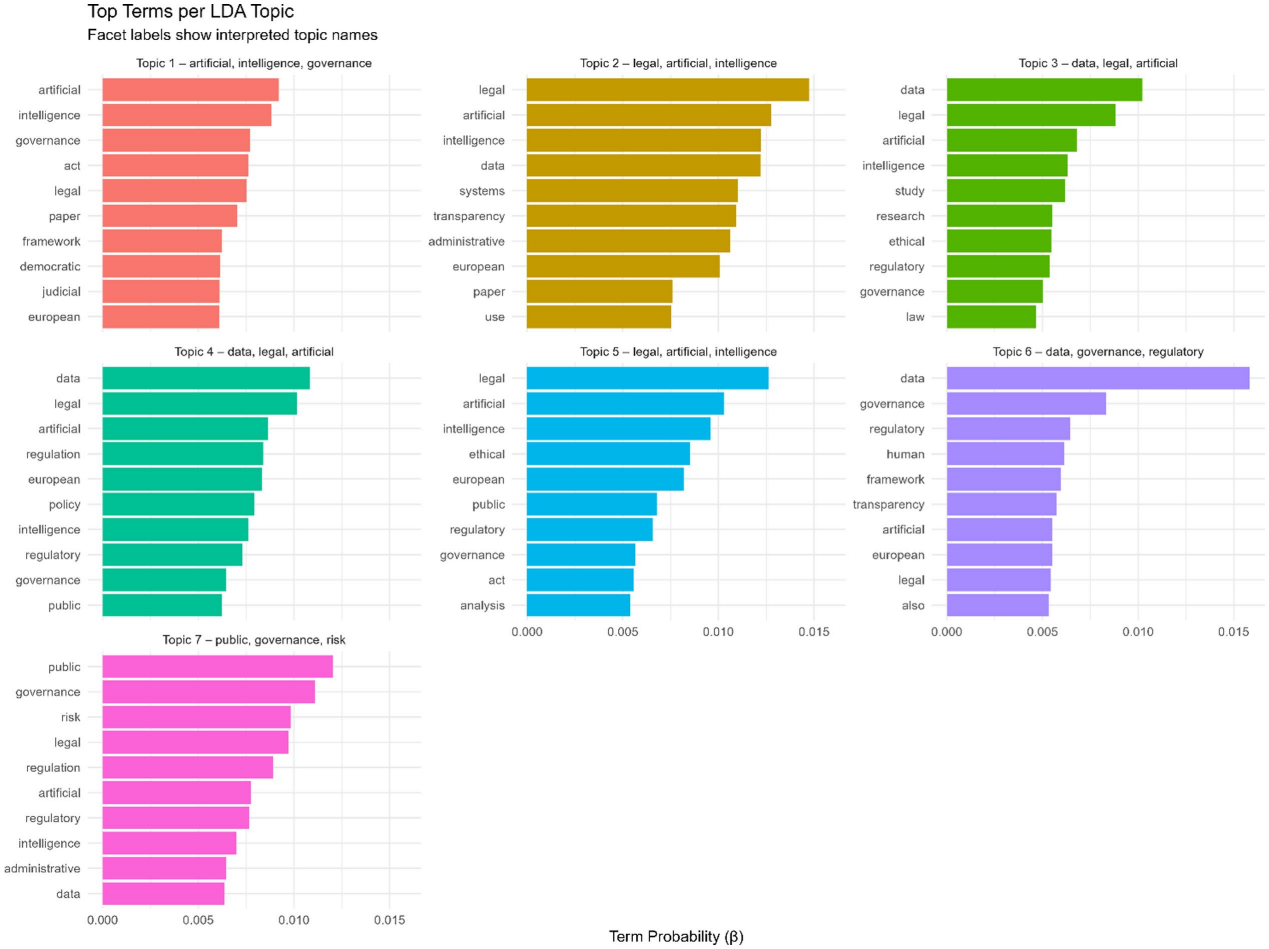
Top terms per LDA topic.

The distribution of top terms across the seven LDA topics (Figure 8) provides a detailed view of the semantic structure underlying AI governance research. Each topic is defined by a coherent cluster of high-probability terms (beta values), indicating the words most representative of that topic. Topic 1 is dominated by terms such as artificial intelligence, governance, legal, and act, reflecting a strong focus on the governance and regulatory architecture surrounding AI systems. Topic 2 has a similar legal orientation but places greater emphasis on systems, transparency, and administrative aspects, suggesting discussions about procedural and institutional mechanisms. Topics 3 and 4 are heavily structured around data and legal terminology, highlighting the centrality of data governance, regulatory compliance, and European policy frameworks. Topic 5 further reinforces the legal-ethical dimension through terms such as ethical, analysis, and public, indicating debates on normative and evaluative aspects of AI oversight. Topic 6 is characterized by a clear emphasis on data, governance, and regulatory concepts, pointing to work on governance infrastructure and transparency mechanisms. Topic 7 clusters around public, governance, and risk, reflecting literature concerned with administrative risk management, public sector decision-making, and regulatory safeguards. Overall, the top terms within each topic confirm that the field is strongly shaped by legal, regulatory, and governance-oriented discourses with consistent emphasis on European policy contexts and data-intensive oversight structures.

**Figure 9:**
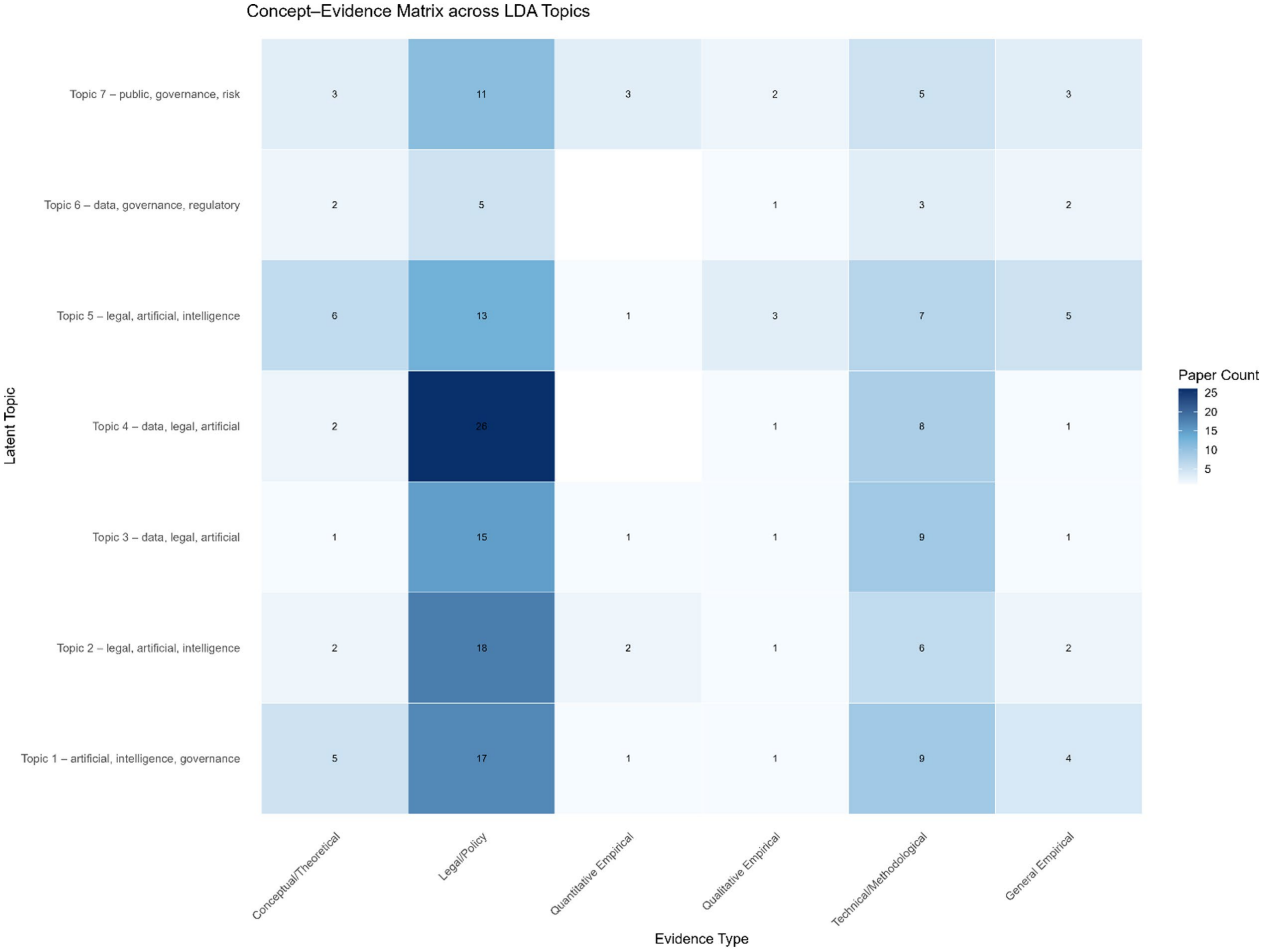
Concept evidence matrix across LDA topics.

The distribution of evidence types across seven LDA-derived topics (Figure 9) shows clear differences in how the field addresses AI governance questions. In all topics, legal and policy analyses predominate, reflecting the central role of regulatory, doctrinal, and institutional perspectives in this research area. Technical or methodological contributions appear consistently but at lower frequencies, indicating that computational, algorithmic, and system-oriented work remains a supporting rather than leading strand. Conceptual and theoretical papers are present in every topic but rarely constitute the majority, suggesting that foundational frameworks are actively used but less frequently extended. Empirical studies, both general and qualitative, occur intermittently, pointing to a limited but growing engagement with applied, data-driven examinations of AI governance phenomena. Notably, Topics 1 and 5 show the most balanced distribution across evidence types, while Topic 4 is overwhelmingly legal and policy oriented. Overall, the configuration indicates a field still anchored in normative and regulatory scholarship, with methodological diversification emerging unevenly across thematic areas.

**Figure 10:**
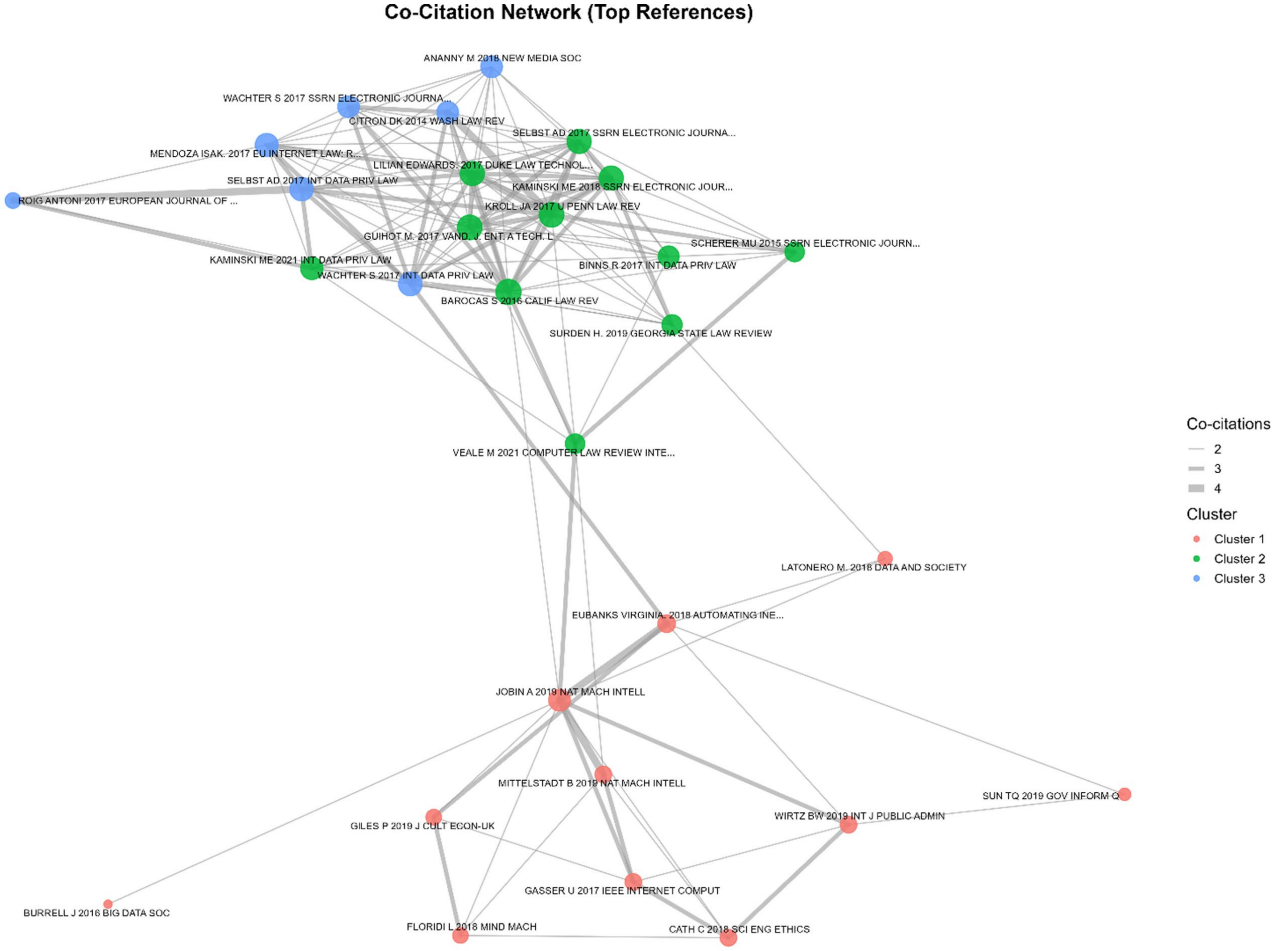
Co-citation network.

The co-citation network of foundational works in AI governance reveals three major clusters, indicating distinct thematic focuses within the field (Figure 10). Cluster 1 (e.g., Eubanks, 2018; Jobin et al., 2019; Mittelstadt, 2019) primarily represents scholarship on the ethical and societal implications of AI, emphasizing automation, inequality, and ethical frameworks. Cluster 2 (e.g., Barocas and Selbst, 2016; Kroll et al., 2017; Veale and Zuiderveen Borgesius, 2021) centers on regulatory and legal scholarship, including discussions of data protection, accountability, and governance mechanisms. Cluster 3 (e.g., Selbst and Powles, 2017; Ananny and Crawford, 2018; Wachter et al., 2021) focuses on technical and policy intersections, highlighting algorithmic accountability, fairness, and practical implications for AI deployment.

Node degree and strength values indicate the relative centrality and influence of specific references within the network. For example, Barocas and Selbst (2016) and Kroll et al. (2017) exhibit high degrees and strengths, suggesting they serve as key hubs bridging legal and technical discussions. Edge weights reflect the frequency with which two references are cited together, with stronger connections observed within clusters, indicating thematic coherence, and weaker ties between clusters, illustrating cross-cutting dialogue. Overall, this network highlights the multidimensional structure of the field, showing both tightly knit subfields and points of interdisciplinary integration.

**Figure 11:**
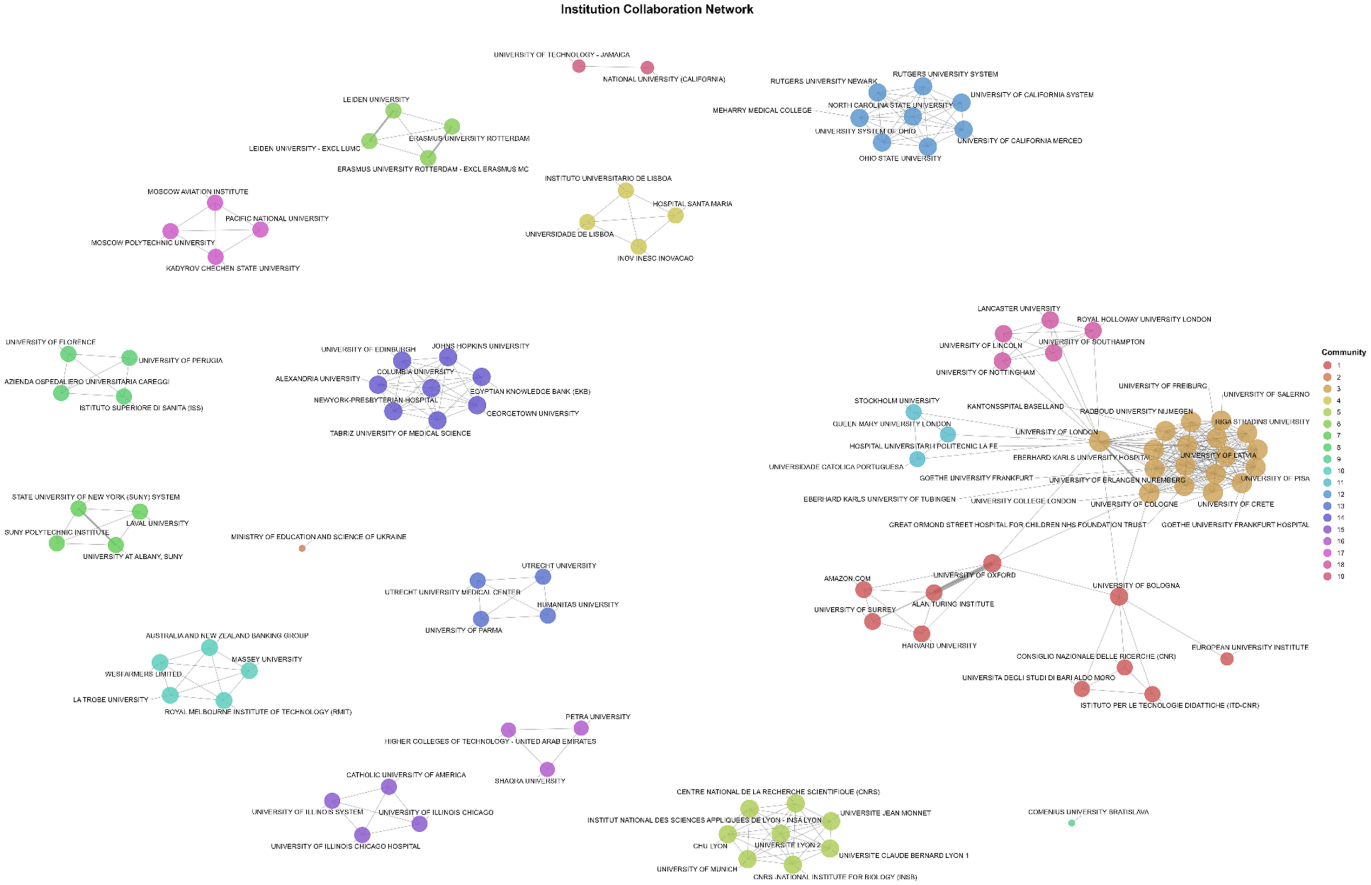
Institution collaboration network.

The institutional collaboration network, based on 209 papers with affiliation data, includes 292 institutions and 1,162 connections. After filtering the most active nodes (100 institutions, 336 links), 19 collaboration communities emerged (Figure 11).

The network is moderately fragmented but internationally connected, with European institutions clearly dominant—particularly the University of London, University College London, University of Pisa, University of Oxford, and University of Freiburg, which form the collaborative core. These hubs anchor clusters extending to Italy, Germany, Portugal, and the Netherlands, while smaller transatlantic partnerships link European universities with counterparts in the United States, Egypt, and Oceania.

Overall, the structure reflects a European-centered research ecosystem with expanding global linkages (Gamerdinger and Willers, 2025). Collaborations are strongest within academic and policy institutions focused on the law, governance, and ethics of AI, suggesting that international cooperation in this field is largely coordinated through European regulatory and academic frameworks. At the country level, these institutional linkages consolidate into broader regional and transnational collaboration patterns, highlighting how research on AI governance is distributed across national systems and policy contexts.

**Figure 12:**
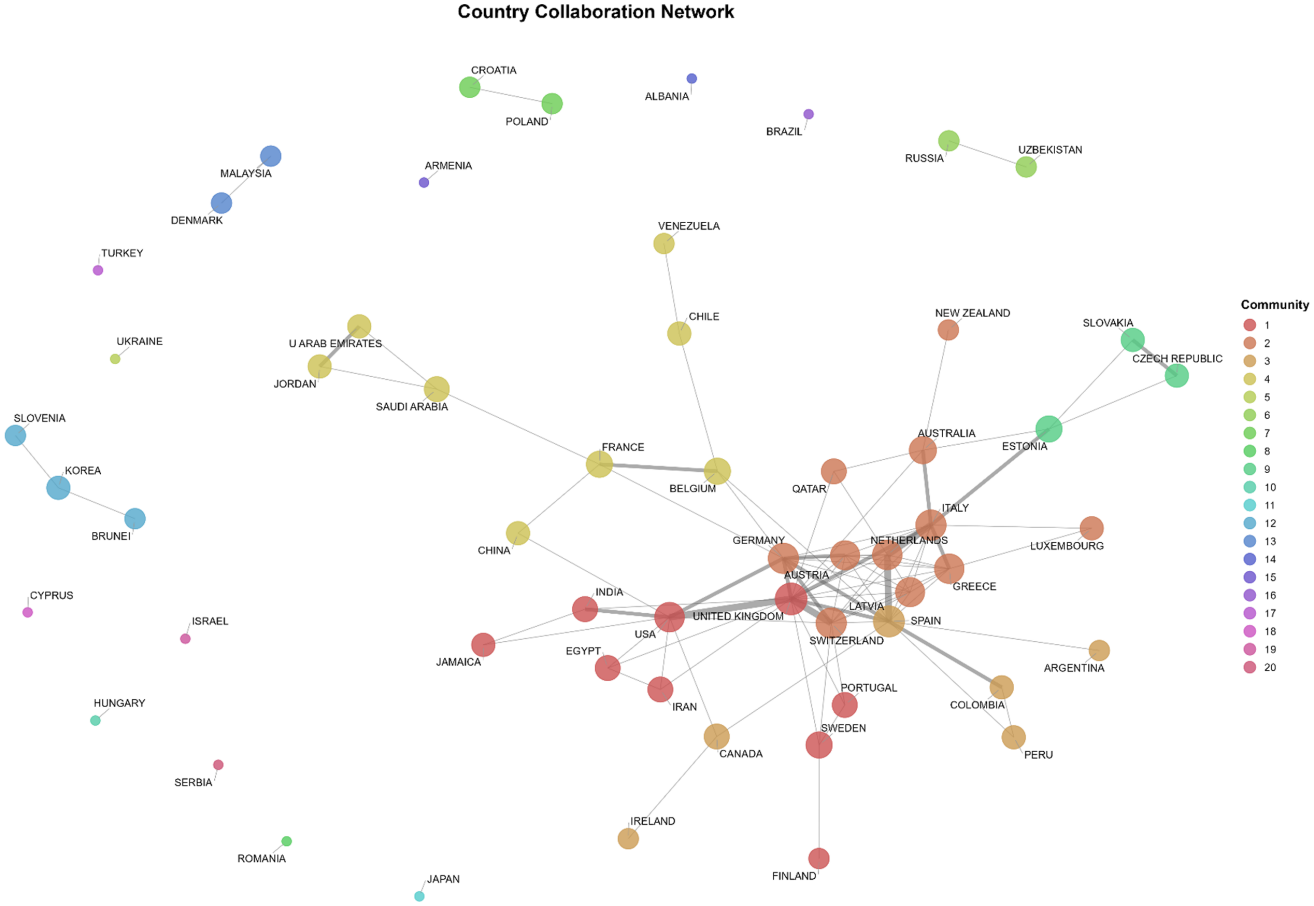
Country collaboration network.

The country collaboration network (Figure 12) consists of 57 nodes and 91 edges, indicating a moderately connected but regionally clustered international research landscape. The most central and collaborative countries are the United Kingdom, Spain, Italy, the United States, and Germany, which serve as key hubs linking otherwise localized research clusters, particularly between European and Anglophone contexts.

Nineteen distinct collaboration communities were identified. The largest clusters include a transatlantic network centered on the United Kingdom, the United States, and Sweden, and a southern European cluster connecting Spain, Italy, and Greece. Smaller, regionally bounded clusters, such as those involving Australia, Estonia, the Czech Republic, France, Saudi Arabia, and Belgium, suggest targeted partnerships rather than broad international integration. Peripheral communities, often composed of single or low-degree countries, such as Ukraine, Japan, or Albania, reflect more limited international engagement and marginal positions within the global network.

In summary, the network displays a clear hierarchical and clustered structure, with a small set of highly connected countries forming a central backbone that facilitates inter-community connectivity, while most other countries remain peripherally linked or isolated. The combined analysis of degree, strength, and community composition reveals a dual pattern of concentrated central hubs alongside regionally bounded clusters, likely shaped by geographic proximity, shared language, or thematic research alignment.

Overall, the science mapping results offer a comprehensive overview of the structural characteristics of the research field, including key actors, international collaborations, and intellectual organization. These patterns reveal a moderately cohesive but thematically diverse research landscape, shaped by a limited number of central hubs and several peripheral or regionally bound contributors.

### 3.3 Trend analysis—temporal evolution of key themes and keywords

The trend analysis explores how research activity and thematic focus have evolved over time, providing insights into the temporal dynamics of scholarly output in the field. By examining author productivity trends and the longitudinal distribution of keywords, this section shows how intellectual attention has shifted, consolidated, or diversified across different periods. This temporal perspective complements the previous science mapping by revealing patterns of growth, continuity, and emerging specialization that characterize the field’s ongoing development.

**Figure 13:**
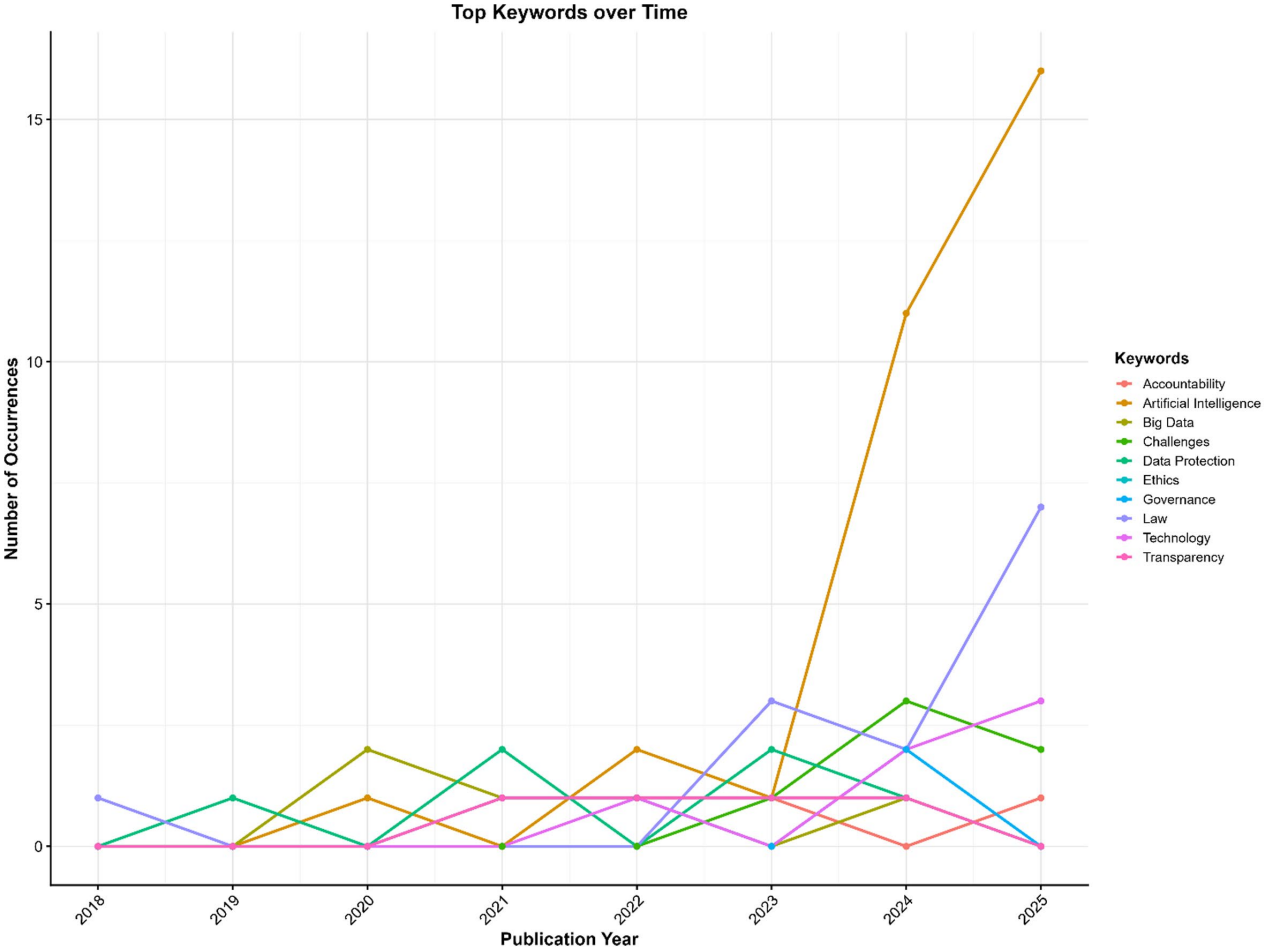
Top keywords over time.

The temporal evolution of the most frequent keywords (Figure 13) reveals a marked concentration around Artificial Intelligence, especially after 2023, reflecting the field’s increasing consolidation and prominence in the broader discussion of AI regulation and governance. Early publications (2018–2021) showed dispersed attention across topics such as Law, Data Protection, and Big Data, with these themes intensifying only gradually. From 2023 onward, the sharp increase in references to Artificial Intelligence, along with the consistent presence of Law, Ethics, and Governance, indicates a thematic convergence toward regulatory and normative aspects of AI systems.

Secondary but recurring terms such as Transparency, Accountability, and Challenges suggest that ethical and procedural considerations remain important, though less dominant, elements of the research agenda. The overall trajectory reflects a shift from broadly technological or data-centric discussions to a mature, policy-oriented research focus centered on legal frameworks and responsible AI governance.

**Figure 14:**
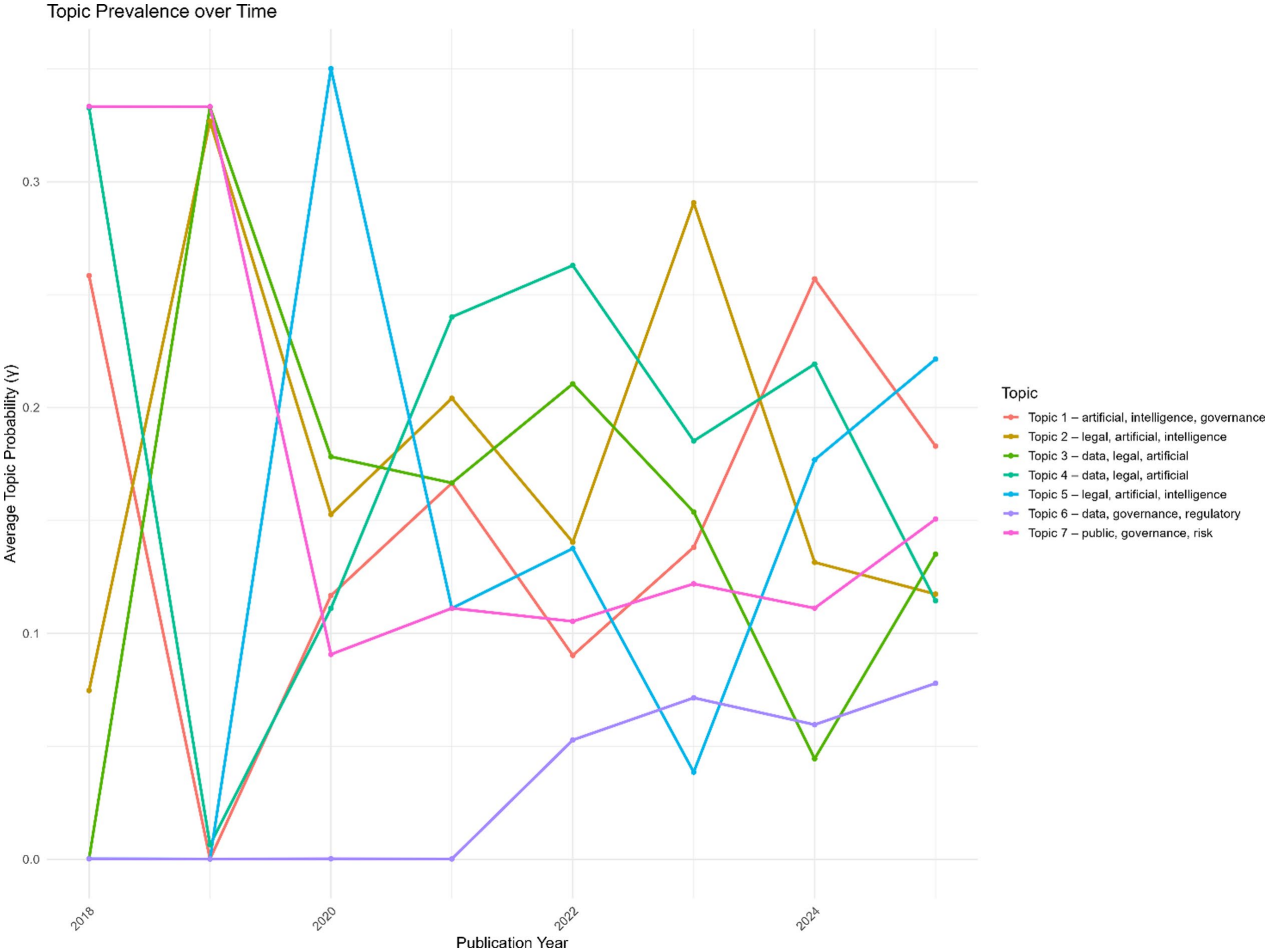
Topic prevalence over time.

The longitudinal analysis of topic prevalence illustrates shifts in research emphasis within AI governance and law between 2018 and 2025 (Figure 14). Topic 1 (artificial, intelligence, governance) shows fluctuating prominence, peaking in 2018 and 2024, reflecting ongoing scholarly interest in governance frameworks. Topics 2 (legal, artificial, intelligence) and 5 (legal, AI-focused) gain traction from 2019 onward, indicating growing attention to the regulatory and legal dimensions of AI. Topics 3 and 4 (data and legal intersections) show moderate but consistent prevalence, suggesting sustained discussion of data governance and compliance issues. Topic 6 (data, governance, regulatory) remains relatively niche but gradually rises, while topic 7 (public governance, risk) maintains steady visibility, underscoring continuous concern with societal and policy implications. Overall, the figure highlights the evolving research landscape, with legal and governance-focused themes increasingly shaping the field alongside core AI governance topics.

The distribution of the most prolific and influential authors shows a clear concentration of high-impact scholarship between 2020 and 2025, with especially strong citation performance among early contributors. Foundational works by Floridi (2018), Kaminski (2019), and Henman (2020) established the intellectual groundwork for later debates on the ethical, regulatory, and governance dimensions of Artificial Intelligence, achieving the highest citation counts (e.g., Henman, 123; Floridi,108; Kaminski, 95) and underscoring their formative role in shaping the field’s conceptual core.

The 2021 cohort, including Ulnicane et al. (2021) and Wachter et al. (2021), marks a peak in productivity and influence, characterized by a surge in multidisciplinary engagement across law, ethics, and technology studies. During 2022–2023, the field entered a transitional phase: foundational and 2021 scholars continued to accumulate citations, while new authors began to join the discourse, expanding the network of contributors and signaling the early stages of broader international and interdisciplinary engagement. This trend accelerates in 2024–2025, with emerging authors such as Cantero Gamito (2024), Cantero Gamito and Marsden (2024), Laux et al. (2024), Migliorini (2024), Bignami et al. (2025a, 2025b), Migliorini and Moreira (2025), and Radanliev (2025a, 2025b), whose current citation counts remain modest due to recency rather than reduced relevance.

Overall, these patterns suggest a generational layering of research influence: early conceptual leaders established the normative and philosophical foundation of AI governance, followed by a second wave of empirical and policy-oriented scholarship. The current expansion reflects a broadening institutional and disciplinary base, characterized by increasing multi-author collaborations and more diverse regional participation.

### 3.4 Validation results: WoS vs. Scopus

To assess the robustness of WoS-based findings, we conducted a cross-validation using the Scopus database, as these are the two most frequently used for such purposes (Liu et al., 2025). We applied the same search query to Scopus, generating a dataset of 298 publications, compared with 209 publications retrieved from WoS. This comparison demonstrates that Scopus provides slightly broader coverage, consistent with previous studies highlighting database-specific differences (Meho and Rogers, 2008; Mongeon and Paul-Hus, 2016; Bar-Ilan, 2018; Singh et al., 2021).

#### 3.4.1 Publication volume and annual growth

The annual distribution of publications between WoS and Scopus shows both similarities and notable differences. Between 2018 and 2022, publication counts in both databases are relatively low and comparable, with Scopus ranging from 2 to 4 articles per year and WoS from 3 to 19. From 2023 onward, the divergence becomes more pronounced: Scopus records a rapid increase from 16 articles in 2023 to 162 articles in 2025, while WoS grows from 26 to 86 articles over the same period. In 2024, Scopus reports 75 articles compared to 45 in WoS. This accelerated growth in Scopus likely reflects its broader coverage of conference proceedings and regional journals. Despite these differences, the overall trend is a substantial increase in research output starting in 2023. This trend is consistent across both databases, supporting the temporal robustness of WoS-based analysis.

#### 3.4.2 Most productive countries

A comparison of country-level productivity showed strong alignment between the two datasets, with some differences in rankings and total article counts. In Scopus, the United States (64 articles), Italy (53), Germany (39), India (37), and the United Kingdom (32) were the top five contributors. In WoS, the United States (49), the United Kingdom (37), Italy (36), Spain (33), and Germany (27) were the leading contributors. While the United States consistently ranks first, Scopus identifies Italy and Germany as slightly more productive than in WoS, likely due to Scopus’s broader coverage of regional journals. This broader regional coverage is significant because Scopus indexes more national and specialty journals (Asubiaro et al., 2024), which can increase publication counts for countries with strong domestic publishing ecosystems—such as Italy and Germany—whose output is more fully captured in Scopus than in Web of Science. Notably, several countries, including Belgium, Greece, and the Netherlands, have higher counts in Scopus than in WoS, indicating potential indexing gaps in WoS for certain regional publications.

#### 3.4.3 Most productive and influential authors

A comparison of author-level productivity between WoS and Scopus shows substantial overlap among leading contributors, while also highlighting some differences due to database coverage and name disambiguation. In bibliometric analysis, leading contributors are the authors, institutions, countries, or journals that produce the most substantial or influential research within a defined field, topic, or period. Their prominence is typically established through indicators such as publication volume, citation impact, h-index values, and co-authorship networks, which together reveal the distribution of knowledge production and influence within a scholarly domain (Aria and Cuccurullo, 2017). Identifying these contributors enables researchers to map a field’s intellectual structure, highlight central knowledge producers, and trace patterns of collaboration and research development (Van Eck and Waltman, 2010). Because these outcomes depend directly on the parameters of the search—particularly the time frame, keywords, and topical boundaries—the design of the search strategy critically shapes the resulting landscape. In this study, the search strategy was tailored to capture literature at the intersection of artificial intelligence, regulation, legal accountability, and public governance.

Authors Floridi, Kaminski, and Radaniev consistently appear among the most productive and cited authors in both databases. For example, Floridi has publications in both 2018 and 2022 in WoS (one article each, with 108 and 31 citations, respectively) and in Scopus (one article each, with 132 and 53 citations, respectively). Similarly, Kaminski has publications in 2019, 2021, and 2023 in both databases, with citation counts reflecting comparable influence.

Some authors are present in only one database due to coverage differences. For instance, De Hert, Henman, and Mittelstadt appear in WoS but are not included in the Scopus dataset, whereas Aloisi, Anagnostopoulos, and Cantero Gamito appear in Scopus but are absent from WoS. Despite these minor discrepancies, the overall trends in productivity and influence are consistent, confirming that WoS-based author-level analyses capture the core contributors in the field. These results further validate the robustness of the primary WoS analysis while highlighting that Scopus can provide complementary coverage, particularly for emerging authors or recent publications.

#### 3.4.4 Leading journals

A comparison of journal-level productivity between WoS and Scopus shows substantial overlap in core publication venues, while also highlighting differences in coverage and citation counts. In WoS, the most productive journals are Computer Law & Security Review (10 articles), European Journal of Risk Regulation (9 articles), and Information & Communication Technology Law (5 articles). In Scopus, the leading journals are Lecture Notes in Networks and Systems (7 articles), CEUR Workshop Proceedings (6 articles), and Computer Law & Security Review (5 articles). Several journals appear exclusively in one database, for example, Lecture Notes in Networks and Systems is among the top in Scopus but does not appear in WoS, whereas European Journal of Risk Regulation ranks high in WoS but has fewer articles in Scopus. Despite these differences, both databases consistently identify Computer Law & Security Review as a central outlet, and the overall pattern of core journals aligns closely. Minor discrepancies reflect differences in indexing policies, coverage of conference proceedings, and regional journals.

#### 3.4.5 Keywords and thematic descriptors

A comparative analysis of the most frequent keywords in WoS and Scopus shows strong thematic convergence, centered on artificial intelligence and its regulatory, legal, and ethical implications. In WoS, the top keywords are Artificial Intelligence, Law, Transparency, Governance, Management, and Data Protection. In Scopus, the most frequent keywords are Artificial Intelligence, European Union, Ethical Technology, Regulatory Compliance, Decision Making, and International Law. Both datasets consistently highlight AI as the central theme, along with governance, ethics, and regulatory frameworks. Differences in ranking reflect the broader coverage of Scopus, which includes additional policy- and EU-focused terms, while WoS emphasizes legal and data protection aspects. Overall, the keyword comparison confirms that both databases represent the same core research domain and provide complementary perspectives on AI regulation and governance.

#### 3.4.6 Summary

Overall, the cross-validation between WoS and Scopus demonstrates strong convergence in core patterns of publication, authorship, journals, and keywords, confirming the robustness of WoS-based analysis. While Scopus offers broader coverage, especially of recent publications, conference proceedings, and EU-focused research, the central themes of artificial intelligence, regulatory frameworks, legal compliance, and ethical considerations are consistently captured in both databases. The alignment of leading authors and journals across datasets further reinforces the representativeness of the WoS corpus, while the keyword comparison highlights complementary perspectives on AI governance, policy, and technology. Together, these results validate the temporal, geographical, and thematic trends identified in the primary analysis and provide a reliable foundation for the subsequent discussion of emerging research directions and gaps in AI regulation and governance.

## 4 Discussion

The research findings reveal a rapidly expanding, thematically diverse, and increasingly collaborative scientific domain, strongly influenced by European regulatory developments. We interpret the bibliometric structures through two complementary perspectives. First, regulatory governance views AI regulation as a mix of instruments that translate public values into specific obligations, standards, risk classifications, auditability requirements, and credible enforcement. In this view, regulation is not merely policy text; it is an institutional architecture that distributes and reallocates responsibilities among developers, deployers, regulators, and adjudicatory bodies through monitoring, inspection, and sanctioning capacities (Black, 2008; Levi-Faur, 2011). Second, public-sector accountability considers governance as a relationship in which actors must explain and justify their conduct to relevant forums, with the possibility of consequences. It recognizes multiple accountability forums and modalities, including legal accountability (liability, compliance, judicial review), political accountability (democratic control), administrative or hierarchical accountability (internal controls, audit, due process), professional accountability (standards of practice), and social accountability (transparency, contestability, rights-based claims) (Mulgan, 2000; Bovens, 2007; Fox, 2007).

The regulation-accountability nexus thus refers to scholarship in which regulatory instruments and enforcement architectures are explicitly used to assign, monitor, and sanction responsibility for AI-enabled decisions in public administration (Romzek and Dubnick, 1987). Using this perspective, bibliometric maps serve as diagnostic evidence: a legally anchored keyword core indicates that the literature’s dominant accountability forum is legal-regulatory, while organizational capacity, implementation constraints, and political accountability mechanisms receive comparatively less theoretical development (Mulgan, 2000). Similarly, a fragmented, faceted network aligns with accountability regimes evolving in domain-specific silos rather than converging into an integrated governance framework that links legal requirements to administrative routines and measurable governance outcomes (Schedler, 1999).

RQ1, which examines global publication trends, is clearly reflected in the steep increase in annual scientific production. The number of publications rose from only a few articles per year in 2018 and 2019 to 86 per year in 2025, demonstrating accelerating scholarly attention to AI regulation and governance. This growth pattern supports the paper’s central design choice: treating 2018–2025 as a distinct governance regime shaped by transformer-enabled capability escalation and the rise of foundation models that generalize across tasks at scale (Vaswani et al., 2017; Bommasani et al., 2021). From a regulatory governance perspective, the post-2020 inflection is consistent with scholarship responding to institutional rulemaking and compliance architectures, as risk-based obligations for AI systems moved from soft principles to formal regulatory proposals and implementation frameworks. From a public accountability perspective, the acceleration also aligns with intensified concern over who is responsible for AI-enabled decisions, through which oversight forums, and with which enforceable remedies, as the literature on algorithmic accountability increasingly centers transparency, contestability, traceability, and accountability-by-design (Diakopoulos, 2016). Isolating this phase provides a clearer, more coherent view of how contemporary AI developments have influenced legal and public sector scholarship—insights that would be diluted in a broader historical frame. Furthermore, this trend is consistent with other bibliometric reviews in similarly regulation-sensitive domains, such as ISO governance in healthcare (Villa-Gallón et al., 2024) and emerging AI subfields (Acim et al., 2025). The clear inflection point after 2020 corresponds to intensified global policymaking, including the EU AI Act, which has evidently stimulated academic output.

RQ2, concerning influential authors, institutions, and countries, is supported by descriptive and network analyses. The most productive countries are the United States, the United Kingdom, Italy, Spain, Germany, and the Netherlands. These findings closely reflect the geographical distribution of regulatory activity and academic capacity in AI governance. The institutional collaboration network identifies the University of London, University College London, University of Pisa, University of Oxford, and the University of Freiburg as central hubs (Figure 11). These institutions anchor the conceptual core of the field, particularly in areas such as fundamental rights, accountability, and administrative law. This institutional clustering aligns with patterns identified by Ali Abaker Omer and Dong (2025), who observed similar central–peripheral dynamics in bibliometric software adoption and methodological transparency research.

RQ3, regarding sources and outlets, is supported by the disciplinary concentration visible in the Web of Science categories used in the sample, which predominantly include Law, Ethics, Computer Science Interdisciplinary Applications, Public Administration, and Political Science. Although the dataset does not include a “top journals” figure, keyword patterns and author affiliations confirm that the field is anchored in policy, legal, and governance journals consistent with its interdisciplinary nature. These outcomes mirror the source patterns found in Publications-journal bibliometric studies (Villa-Gallón et al., 2024; Acim et al., 2025).

RQ4, which concerns citation dynamics, is reflected in the observation that older publications (2018–2021) have the highest citation counts, indicating that these early works have shaped foundational debates in AI governance. The declining average citations for more recent years reflect the normal citation lag in fast-growing fields. The co-occurrence relationships in the keyword network (Figures 5, 6) further demonstrate intellectual consolidation around law, transparency, data protection, governance, and accountability concepts that frequently co-appear and structure the conceptual landscape.

RQ5, which examines scientific collaborations, is strongly supported by institutional and country networks. The institutional network (Figure 11) reveals a moderately cohesive set of collaborations dominated by European universities, with transnational links to the United States and Australia. The country network (Figure 12) identifies the United Kingdom, Spain, Italy, Germany, and the United States as key nodes. Nineteen distinct collaboration communities indicate that the field is globally dispersed but regionally anchored, consistent with the regulatory ecosystems shaping research agendas. These findings parallel patterns observed in other AI-related bibliometric landscapes, such as generative AI and deepfakes (Acim et al., 2025).

RQ6, which addresses emerging themes, is clarified by keyword analyses. The most frequent keywords—Artificial Intelligence, Law, Transparency, Governance, Management, Data Protection, and Technology (Figure 2)—show that the field is legally anchored and governance oriented. Viewed through an accountability lens, the prominence of Law, Transparency, Data Protection, Governance, and Accountability indicates that the literature operationalizes AI governance primarily through legal accountability and compliance logics—such as duties, procedural safeguards, reviewability, and enforceable consequences—rather than through administrative capacity building and internal management controls (May, 2007). The distribution of LDA topics in our results aligns with this orientation, as legal and policy analyses dominate across themes, while empirical work and explicit theory extension are comparatively secondary. This pattern tends to reproduce parallel, partly incompatible accountability conceptions across domains (Koppell, 2005; Mashaw, 2006). This helps explain why the field can be conceptually rich yet weakly cumulative: accountability traverses multiple sectoral and legal settings but lacks a shared public administration mechanism that links external regulatory requirements to implementation routines, street-level practice, and measurable governance outcomes (Hupe and Hill, 2007). The keyword co-occurrence network (Figure 4) reveals clusters connecting AI to core regulatory topics such as accountability, privacy, ethics, and risk. The thematic map (Figure 3) identifies Accountability, Human Rights, and Impact as motor themes, meaning they are both central and internally developed. Basic themes with high centrality, but lower density include Artificial Intelligence, Governance, and Law, confirming that these topics serve as cross-cutting concepts integrating the field. Emerging or less developed themes include Autonomous Vehicles, Adoptions, and Rights, indicating future research areas that have not yet been fully consolidated. The faceted network (Figure 6) further highlights thematic fragmentation into seven domain-specific clusters, confirming that AI governance research remains interdisciplinary but not yet fully integrated.

RQ7, which addresses research gaps, emerges directly from the thematic and cluster analyses. Underdeveloped areas include AI-related procedural safeguards, enforcement capacity, complex administrative decision-making, and cross-border governance. The weak connections between clusters indicate that research on technical systems, legal frameworks, and organizational practices remains siloed. These gaps reflect concerns identified in comparative bibliometric analyses (Ali Abaker Omer and Dong, 2025), which highlight methodological fragmentation in interdisciplinary fields. Additionally, the limited presence of themes related to administrative capacity, legitimacy, and institutional complexity indicates a need for deeper integration of public administration and legal theory with computer science approaches. This also suggests that policymakers should strengthen institutional capabilities for AI governance and try to ensure that AI systems align with existing administrative procedures and structures.

Taken together, the findings provide a coherent and structured understanding of how academic discourse on AI, regulation, and public governance has evolved during a period of intense technological advancement and regulatory institutionalization. This study’s results are consistent with emerging bibliometric evidence in related AI governance domains (Villa-Gallón et al., 2024; Acim et al., 2025) and extend this work by offering focused insights on legal accountability and public sector governance. By identifying dominant clusters, collaboration networks, and thematic gaps, the analysis clarifies how the scholarly landscape is organized and where further interdisciplinarity and empirical grounding are needed. As regulatory frameworks mature and AI becomes increasingly embedded in public administration, future research should connect fragmented thematic clusters, particularly by bridging legal accountability, organizational implementation, and empirical governance outcomes.

## 5 Implications

The results of this study have several important implications for academic research, policymaking, and institutional practice in the governance of artificial intelligence.

First, the sharp rise in publications after 2022 indicates that regulatory initiatives, particularly the EU AI Act, have become key drivers of scholarly activity. This demonstrates that AI governance research responds to institutional developments and regulatory milestones rather than evolving independently. Researchers and policymakers should view academic literature as both a reflection of regulatory shifts and a resource for shaping future governance frameworks.

Second, the concentration of scientific production in Europe and among a small number of leading institutions highlights both strengths and vulnerabilities in the global research landscape. While European institutions provide intellectual leadership, the limited representation of regions outside Europe and North America suggests an imbalance that may hinder the development of globally relevant regulatory perspectives. These findings underscore the need for funding bodies and international organizations to foster more geographically inclusive research collaborations.

Third, the thematic analysis shows that AI governance research is anchored in legally oriented concepts that connect technical system design with public-sector accountability. This creates opportunities to advance hybrid approaches that combine AI system architecture, administrative law, and public management. Similar calls for integrating technical and governance perspectives appear in recent bibliometric analyses in other fields (Villa-Gallón et al., 2024; Ali Abaker Omer and Dong, 2025). Policymakers and research institutions should support collaborative work that bridges legal, ethical, and computational perspectives.

Fourth, the identification of underdeveloped themes—such as legitimacy, institutional complexity, and rights implications for automated decision-making—indicates areas where scholarly attention remains limited despite their importance for public governance. Legitimacy, institutional complexity, and rights implications underscore that the use of AI in public administration is not merely a matter of optimizing service delivery but also one of cultivating citizen trust and acceptance through transparency, appropriate human oversight, and alignment with public expectations. At the same time, institutional complexity further complicates AI adoption because mismatches between established administrative norms and the logics embedded in algorithmic systems can create gaps between policy design and citizen preferences, a dynamic empirically demonstrated in studies examining legitimacy perceptions among public administrators and citizens (Grimmelikhuijsen and Meijer, 2022; Hillo et al., 2025). Strengthening these research areas could improve the design of AI oversight mechanisms, enhance fundamental rights protections, and support responsible implementation of AI in public administration.

To link the policy relevance, the findings highlight the need to establish policies that strengthen the institutional capabilities required to govern AI systems, and the necessity for establishing policies that embed transparency, accountability, and participatory mechanisms directly into AI deployment processes to maintain public trust and compliance with democratic norms. Policymakers should also assess how AI interacts with existing administrative procedures, legal constraints, and multilayered decision structures.

## 6 Future research directions

The results of this study highlight several avenues for future research that can strengthen the evidence base on AI governance and improve the design of regulatory and institutional frameworks.

First, underdeveloped themes such as legitimacy, institutional logic, and rights implications require deeper theoretical and empirical investigation. These areas are critical for understanding the societal and democratic consequences of automated decision-making in the public sector.

Second, more empirical work is needed on the implementation of AI systems in real administrative contexts. Existing scholarship remains heavily conceptual, with limited analysis of organizational capacity, enforcement mechanisms, and the operationalization of accountability in public-sector workflows.

Third, future research should examine the effects of the EU AI Act and similar regulatory initiatives on institutional behavior, administrative processes, and cross-border governance. Longitudinal tracking of publication trends, thematic evolution, and collaboration networks will clarify how regulatory frameworks shape scholarly attention and research agendas.

Fourth, international collaborations should be strengthened, especially in underrepresented regions. Expanding cross-regional comparative studies will help address gaps in global governance capacity and ensure that the development of AI oversight does not become overly concentrated in high-income democratic countries.

Fifth, emerging technological developments in multimodal AI and foundation models deserve explicit attention. These technologies are increasingly used in high-stakes public-sector decisions but remain only marginally represented in current thematic clusters. Research should explore how such systems interact with administrative law principles, risk classification schemes, transparency requirements, and institutional accountability. In this context, future research could incorporate multiple data sources and integrate qualitative insights into the data.

Finally, future studies should integrate bibliometric methods with qualitative, doctrinal, or empirical approaches. Mixed-method designs would allow deeper exploration of the practical, ethical, and legal implications of AI deployment in public-sector governance, complementing the structural insights gained from bibliometric analysis.

## 7 Conclusion

This paper presents a comprehensive bibliometric analysis of scientific research on artificial intelligence, regulation, legal accountability, and public governance published between 2018 and 2025. Using data retrieved from the Web of Science Core Collection and employing Bibliometrix and complementary R-based analytical tools, the study systematically maps global publication trends, influential contributors, collaboration networks, and thematic developments in a field increasingly shaped by rapid technological evolution and regulatory transformation.

The results show a clear acceleration of research activity during the examined period, with publication output rising sharply after 2022. This temporal pattern closely aligns with major regulatory developments, most notably the emergence and finalization of the EU AI Act, which appears to have stimulated scholarly attention across law, computer science, ethics, and public governance. The geographic and institutional distribution of research further underscores Europe’s central role in shaping academic debates on AI governance, with the United Kingdom, Italy, Spain, Germany, and the Netherlands forming the intellectual core of the field, supported by globally recognized institutions such as University College London, the University of Oxford, the University of Pisa, and the University of Freiburg.

Citation analyses confirm the foundational influence of earlier publications (2018–2021), which established the conceptual and normative framing of AI regulation and administrative accountability. The thematic analyses reveal a field anchored in legally oriented concepts including law, transparency, governance, accountability, and data protection. Motor themes such as accountability, human rights, and impact represent central, well-developed areas of research, while basic themes such as artificial intelligence, governance, and technology provide cross-cutting conceptual connections across disciplines. Emerging themes related to autonomous systems, rights, legitimacy, and institutional logics indicate areas of growing importance that remain underdeveloped.

The collaboration networks reveal a moderately cohesive, but geographically concentrated research ecosystem. Nineteen collaboration communities show increasing international engagement, with particularly strong ties within Europe and significant connections to the United States and Australia. These patterns indicate that AI governance has become an important global research priority, although contributions from underrepresented regions remain limited.

The analysis shows that research on AI governance, while rapidly developing field, remains thematically fragmented across domain-specific areas. Although the field is interdisciplinary in nature, with technical, legal, and ethical dimensions closely interwoven, ongoing efforts are needed to bridge conceptual gaps between AI innovation, regulatory frameworks, and governance practices.

Overall, the bibliometric map provides a solid foundation for understanding how debates on AI governance are structured and where research capacity should be strengthened to support effective, trustworthy, and accountable AI deployments in public-sector contexts. The results of this study have important implications for research, policy, and practice. For researchers, the thematic fragmentation and underdeveloped clusters highlight critical opportunities for deeper theoretical integration across law, public administration, and technical AI research. For policymakers, the concentration of scholarship around themes such as accountability, transparency, and data protection provides a strong basis for evidence-informed governance but also underscores the need for capacity building in jurisdictions where AI deployment is outpacing regulatory expertise. For practitioners, the findings emphasize the urgency of operationalizing accountability mechanisms, developing administrative oversight capacities, and strengthening institutional readiness for AI implementation.

## Data Availability

The datasets presented in this article are not readily available because the data are part of an ongoing study. Requests to access the datasets should be directed to the authors of the article.
